# The Fim and FhaB adhesins play a crucial role in nasal cavity infection and *Bordetella pertussis* transmission in a novel mouse catarrhal infection model

**DOI:** 10.1371/journal.ppat.1010402

**Published:** 2022-04-08

**Authors:** Jana Holubova, Ondrej Stanek, Attila Juhasz, Illiassou Hamidou Soumana, Peter Makovicky, Peter Sebo

**Affiliations:** 1 Institute of Microbiology of the Czech Academy of Sciences, Prague, Czech Republic; 2 Department of Infectious Diseases, College of Veterinary Medicine, University of Georgia, Athens, Georgia, United States of America; 3 Institute of Molecular Genetics of the Czech Academy of Sciences, Czech Centre for Phenogenomics, Vestec, Czech Republic; INSERM U1019; CNRS UMR 8204; Univ Lille Nord de France; Institut Pasteur de Lille, FRANCE

## Abstract

Pulmonary infections caused by *Bordetella pertussis* used to be the prime cause of infant mortality in the pre-vaccine era and mouse models of pertussis pneumonia served in characterization of *B*. *pertussis* virulence mechanisms. However, the biologically most relevant catarrhal disease stage and *B*. *pertussis* transmission has not been adequately reproduced in adult mice due to limited proliferation of the human-adapted pathogen on murine nasopharyngeal mucosa. We used immunodeficient C57BL/6J MyD88 KO mice to achieve *B*. *pertussis* proliferation to human-like high counts of 10^8^ viable bacteria per nasal cavity to elicit rhinosinusitis accompanied by robust shedding and transmission of *B*. *pertussis* bacteria to adult co-housed MyD88 KO mice. Experiments with a comprehensive set of *B*. *pertussis* mutants revealed that pertussis toxin, adenylate cyclase toxin-hemolysin, the T3SS effector BteA/BopC and several other known virulence factors were dispensable for nasal cavity infection and *B*. *pertussis* transmission in the immunocompromised MyD88 KO mice. In contrast, mutants lacking the filamentous hemagglutinin (FhaB) or fimbriae (Fim) adhesins infected the nasal cavity poorly, shed at low levels and failed to productively infect co-housed MyD88 KO or C57BL/6J mice. FhaB and fimbriae thus appear to play a critical role in *B*. *pertussis* transmission. The here-described novel murine model of *B*. *pertussis*-induced nasal catarrh opens the way to genetic dissection of host mechanisms involved in *B*. *pertussis* shedding and to validation of key bacterial transmission factors that ought to be targeted by future pertussis vaccines.

## Author summary

Pertussis is a strictly human respiratory infectious disease that can be fatal to young children and elderly. The currently used mouse models of intracerebral or pulmonary *B*. *pertussis* infection served remarkably well in identification of *B*. *pertussis* virulence factors and development of efficacious pertussis vaccines. However, *B*. *pertussis* transmission could not be reproduced in adult mice due to limited proliferation of the human pathogen in the upper airways of mice. Therefore, we inoculated nasal cavities of immunodeficient MyD88 knock-out mice to achieve a human-like high level of nasal mucosa infection. This allowed triggering of rhinitis and catarrhal shedding of bacteria from mouse nasal cavity and efficient transmission of the infection onto co-housed adult animals. Testing a set of bacterial mutants, we identified two bacterial adhesins as key transmission factors. Combined with the power of mouse and bacterial genetics, this newly established mouse model of the catarrhal phase of the whooping cough disease will enable deciphering of mechanisms that underlie *B*. *pertussis* transmission to new hosts.

## Introduction

The Gram-negative coccobacilli *Bordetella pertussis* and the human-adapted *B*. *parapertussis*_*HU*_ infect ciliated epithelia of upper airways and elicit a highly contagious human respiratory illness known as pertussis, or whooping cough [1,2]. Pertussis starts by a common cold-like nasopharyngeal catarrh without fever, accompanied by uncontrollably running nose, coryza-elicited sneezing and postnasal drip-elicited cough that results from mechanical irritation of larynx and enables transmission of bacteria in expelled aerosol droplets. The catarrhal disease lasts for about 2 to 3 weeks, over which *B*. *pertussis* proliferates to densities of up to 10^8^ bacterial cells per ml of undiluted nasal aspirate [3]. The pathogen thereby achieves very high transmission rates that peak around two weeks from the onset of cough [4]. The bacteria eventually infect the tracheobronchial tree, provoke accumulation of a thick mucus layer and trigger a progressively intensifying paroxysmal cough disease characterized by high-pitched inspiratory whoops, often lasting for several months. In young children the eventual infection of lungs by the pertussis toxin (PT)-producing *B*. *pertussis* species often yields a life-threatening pneumonia that is frequently complicated by secondary infections. Therefore, pertussis used to be the prime cause of infant mortality prior to introduction of efficient whole cell pertussis (wP) vaccines some seven decades ago [5,6].

Despite high global vaccine coverage, pertussis is the least-controlled vaccine-preventable infectious disease and it is estimated that ~24 million whooping cough cases and ~160,000 pertussis-related deaths occur annually world-wide [7]. Moreover, pertussis outbreaks recently resurged in most developed countries that switched to the use of less reactogenic acellular pertussis (aP) vaccines that confer a shorter-lasting protection from symptomatic infection [8,9] and fail in restricting *B*. *pertussis* transmission [10]. Recent experiments in mice indicated that priming by aP vaccines may even compromise the induction of *B*. *pertussis*-specific IL-17/IFN-γ-secreting tissue-resident memory CD4^+^ T cells that orchestrate the clearance of *B*. *pertussis* from nasopharyngeal mucosa [11–15]. Indeed, circulation of *B*. *pertussis* continues to rise and asymptomatic infections and undiagnosed pertussis appears to be frequent in highly aP-vaccinated populations [8,9,16], which calls for development of vaccines preventing *B*. *pertussis* transmission.

*B*. *pertussis* is a human-adapted pathogen that is particularly well armed for immune evasion [2]. It produces an array of virulence factors, including several adhesins, such as the filamentous hemagglutinin (FhaB processed to FHA), the type I pilus fimbriae (Fim2/3), pertactin (Prn), or the tracheal colonization factor A (TcfA). The bacterium expresses several complement resistance factors, such as the BrkA and Vag8 autotransporters and in particular, it secretes two potent immunosubversive protein toxins. The more notoriously known pertussis toxin (PT) belongs to the AB_5_ toxin family and exerts both local and systemic effects [17]. PT action delays neutrophil recruitment onto the infected mucosa and induces leukocyte proliferation and naïve lymphocyte egress from lymphoid organs and bone marrow, thus eliciting the hyperleukocytosis characteristic of pertussis disease [18]. PT promiscuously invades various host cells and through ADP-ribosylation of the Gα_i/o_ subunits of trimeric G proteins the action of PT hijacks a whole array of GPCR-activated cellular signaling pathways [17], perturbing also induction of adaptive immune responses [19,20]. The other potent immunosuppressive toxin secreted by *B*. *pertussis* is an RTX family adenylate cyclase toxin-hemolysin (AC-Hly, or CyaA) [21]. CyaA primarily penetrates and disarms the complement receptor 3 (CR3)-expressing CD11b^+^ sentinel phagocytes [22,23] and swiftly disrupts their bactericidal functions through unregulated conversion of cellular ATP to the key signaling molecule cAMP [24]. This annihilates the oxidative burst capacity of neutrophils, blocks complement-mediated opsonophagocytic uptake and killing of bacteria [21,25–28], inhibits differentiation of inflammatory monocytes into bactericidal macrophages [29] and triggers airway macrophage apoptosis [24,30,31]. Moreover, CyaA also compromises induction of adaptive T cell immune responses through hijacking of dendritic cell maturation and by inhibiting antigen presentation to T cells [32].

*V*arious mouse strains and aerosol or intranasal inoculation routes were used to develop models of *B*. *pertussis* lung infection. These models enabled discovery of bacterial virulence factors and deciphering of immune responses to infection, exploration of pertussis-related pneumonia pathogenesis and testing of pertussis vaccines [33]. However, the mechanisms and virulence factors underlying the biologically most relevant process of *B*. *pertussis* transmission during the catarrhal phase of pertussis disease remained unexplored due to lack of a useful animal model. Only recently the full course of human pertussis, including catarrhal shedding and transmission by aerosol, could be reproduced in the olive baboon *Papio Anubis* [34,35]. However, this demanding non-human primate model is accessible only at two specialized facilities in the US and the EU, and the limited numbers of available animals and associated costs remain prohibitive. Intriguingly, *B*. *pertussis* transmission between neonatal mice was recently observed [36], but the catarrhal disease and *B*. *pertussis* transmission could not be reproduced in adult mice, due in part to mouse-specific microbiota that confers an enhanced colonization resistance onto murine airway mucosa [37].

We reasoned that *B*. *pertussis* proliferation in mouse upper airways might be restricted by vigorous mobilization of innate antibacterial defense mechanisms upon recognition of *B*. *pertussis*-associated molecular patterns by Toll-like receptors (TLRs) of epithelial and sentinel myeloïd cells of the airway [38]. Therefore, we used mice deficient in TLR signaling and show that a human-like high level of *B*. *pertussis* colonization of murine nasal cavity triggers rhinosinusitis and bacterial shedding, which enables robust *B*. *pertussis* transmission between adult MyD88 knock-out mice and the identification of involved *B*. *pertussis* transmission factors.

## Results

### Restricted proliferation of *B*. *pertussis* in the nasal cavity of immunocompetent mice limits bacterial shedding and transmission

**Fig 1:**
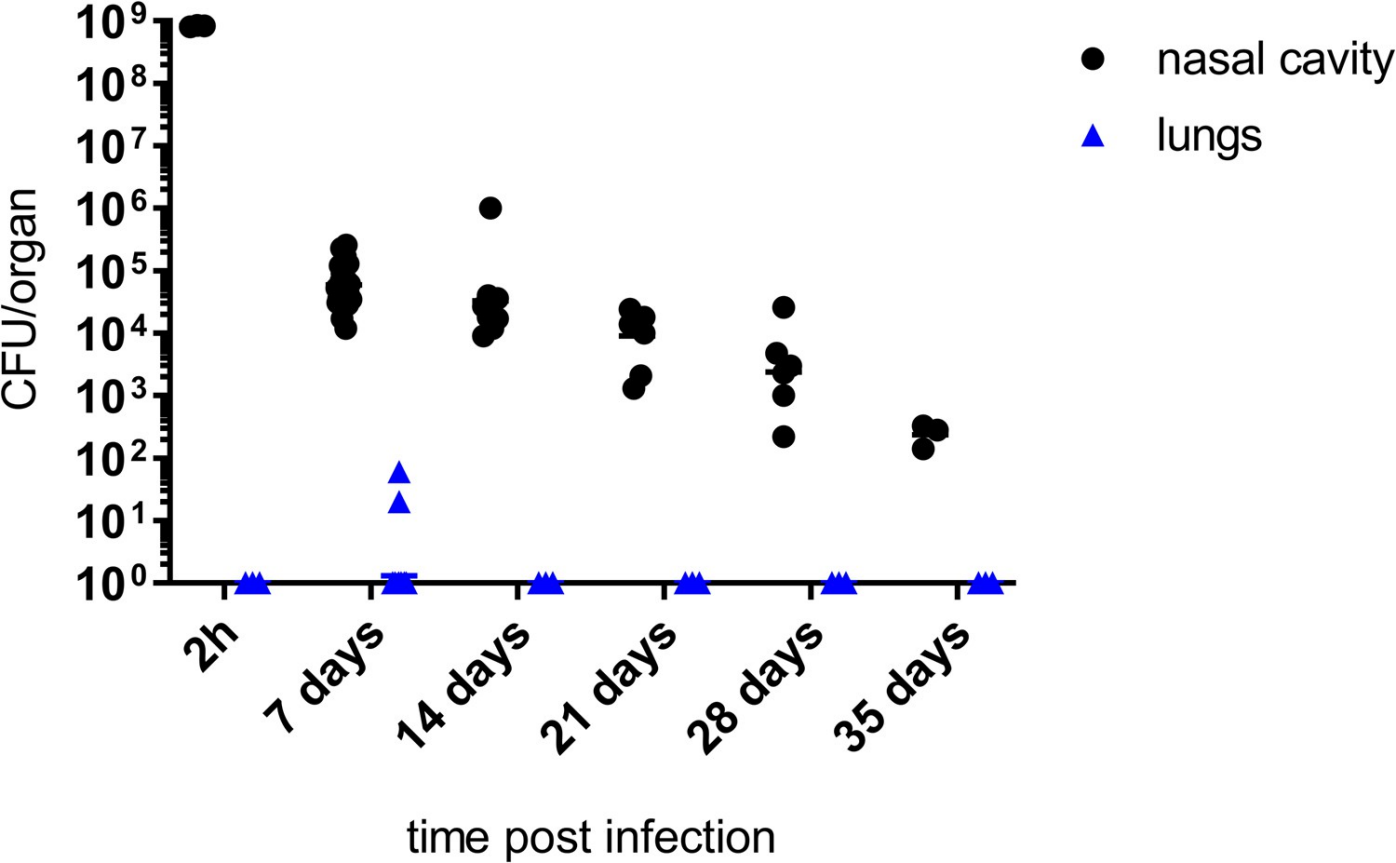
Nasal mucosa of immunocompetent C57BL/6J mice restricts proliferation of *B*. *pertussis* inoculated at a high dose. Nares of C57BL/6J mice were inoculated with 10^9^ CFU of *B*. *pertussis* Tohama I (Str^R^) applied in 5 μl of suspension to prevent inhalation into lungs. At indicated time points the mice were sacrificed and bacterial loads in the nasal cavity and in the lungs were determined by plating of organ homogenates on BG agar. Three mice per time points of 2 h and 35 days were used and groups of 8 mice were used for other time points. Data for day 7 represent a pool of CFU values from 35 mice used in seven independent experiments (n = 35). The horizontal bars indicate the geometric mean values.

We reasoned that *B*. *pertussis* transmission between adult mice was primarily hampered by the restricted proliferation of the human-adapted bacteria within mouse nasal cavity. Therefore, we tested if nasal cavity inoculation by *B*. *pertussis* suspensions reaching bacterial densities found in nasal aspirates from human pertussis patients [3] would overcome the reported colonization resistance of mouse nasal mucosa [37]. To prevent a lethal pulmonary infection due to inhalation of high bacterial numbers (LD_50_ ~3 x 10^7^ CFU), only 5 μl of concentrated bacterial suspensions were applied into the nares of C57BL/6J mice. Such low volumes of tracer suspensions were previously found to be quantitatively retained within mouse nasal cavity [39]. Indeed, when 10^9^ CFU of the streptomycin resistant (Str^R^) variant of *B*. *pertussis* Tohama I were inoculated into mouse nares within 5 μl of SS medium, the infection was largely restricted to the nasal cavity. Only 2 out of 35 inoculated mice exhibited any *B*. *pertussis* infection of the lungs (10^1^–10^2^ CFU) on day 7 (Fig 1), hence at a time point when the lung infection by *B*. *pertussis* usually peaks upon intranasal inoculation with larger (20–50 μl) volumes of bacterial suspensions [40]. Over the follow-up for 35 days a steep initial decrease of *B*. *pertussis* counts from the administered dose of 10^9^ CFU down to ~10^5^ CFU per nasal cavity was observed within the first 7 days of infection, followed by a slower progressive decrease of the nasal CFU counts (Fig 1). Hence, the application of a very high bacterial dose (10^9^ CFU) did not allow to establish a stable nasal cavity colonization level exceeding ~5 x 10^5^ *B*. *pertussis* CFU.

**Fig 2:**
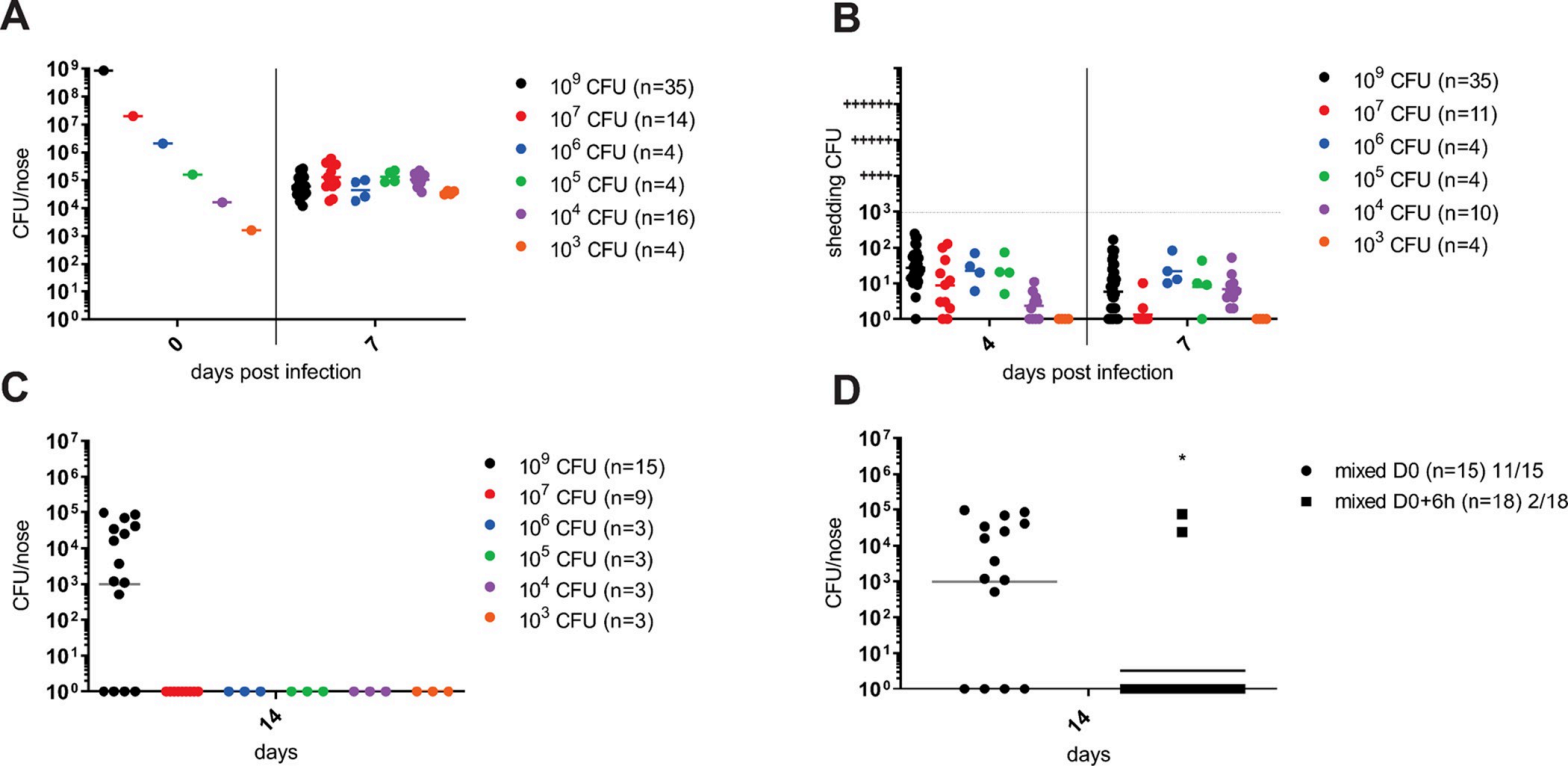
High bacterial inoculation enables low level nasal shedding and occasional transmission of *B*. *pertussis* between adult C57BL/6J mice. (**A**) Nares of C57BL/6J mice were inoculated with 5 μl of bacterial suspensions containing from 10^3^ to 10^9^ CFU of *B*. *pertussis* cells and bacterial loads in infected nasal cavities were determined on day 7 by plating of nose homogenates. (**B**) Prior to euthanasia, *B*. *pertussis* shedding from the nasal cavities of infected mice was quantified on days 4 and 7 by gentle tapping of the mouse noses on the surface of a BG agar plate containing streptomycin (100 μg/ml) and spreading of the deposited bacteria in 100 μl PBS over the plate surface for CFU determination. (**C**) Index mice inoculated into nares with the indicated dose of *B*. *pertussis* CFUs were immediately co-housed in the same cage in a 1:1 ratio with non-inoculated recipient mice for 7 days before the index mice were withdrawn for determination of nasal shedding and euthanized for determination of bacterial loads in their nasal cavities. The co-housed recipient mice were kept for another week prior to determining the bacterial loads in their nasal cavities on day 14. (**D**) In parallel, further index mice inoculated with the highest dose of 10^9^ CFU of *B*. *pertussis* were kept in separate cages for 6 hours before being brought in contact and co-housed in a 1:1 ratio over 7 days with the non-infected recipient mice and nasal shedding, nose colonization level and transmission to recipient mice was analyzed as above. Statistical significance of CFU count difference of transmission between the immediately co-housed mice and mice co-housed with 6 hours of delay was assessed by unpaired t-test. * (p < 0.05). Each dot represents bacterial counts for an individual mouse. Horizontal bars indicate the geometric means. The data for nasal colonization (A) and shedding (B) of the index mice inoculated with 10^9^ CFU were pooled for the 35 mice used in seven independent experiments (n = 35). The number of mice used for each infection dose is given in brackets.

When C57BL/6J mouse nares were inoculated with a series of doses spanning 6 orders of magnitude of CFU administered in 5 μl of suspension, a consistent final level ranging between 10^4^ and 10^6^ *B*. *pertussis* CFU was recovered from mouse nose homogenates on day 7 after infection. Interestingly, the mean of CFU counts upon inoculation with only 10^3^ CFUs increased in 7 days of infection by almost two orders of magnitude. Hence, the low inoculum proliferated in the nasal cavity up to the CFU counts resulting from inoculation with orders of magnitude higher bacterial doses (Fig 2A). These results indicate a threshold of ≤10^6^ *B*. *pertussis* cells that could establish in the nasal cavity of conventional C57BL/6J mice.

We next assessed if this level of mouse nose colonization triggered bacterial shedding on days 4 and 7 after infection by a range of *B*. *pertussis* doses. The tips of noses of inoculated mice were gently tapped 4-times onto selective BG agar plates containing 15% sheep blood and 100 μg/ml of streptomycin and the recovered bacteria were spread over the plate in 100 μl of PBS to enable counting of hemolytic and streptomycin-resistant *B*. *pertussis* colonies formed within 5 days of growth. As shown in Fig 2B, on days 4 and 7 after inoculation by doses differing by several orders of magnitude, all infected C57BL/6J mice shed comparably few *B*. *pertussis* bacteria, with 0 to 100 (mean ~50) CFUs recovered on the BG plates.

To further assess if such inoculated index C57BL/6J mice could transmit the infection onto non-inoculated cage mates, two or three non-inoculated (uninfected recipient) mice were co-housed with 2 or 3 infected index (infected donor) mice at a 1:1 ratio for 7 days. Thereafter, the index mice were withdrawn from the cage and shedding of bacteria from their noses was assessed prior to euthanasia and determination of *B*. *pertussis* counts in nose homogenates. The recipient animals were kept in the cage for another week and sacrificed for determination of bacterial loads in the nasal cavity on day 14. As shown in Fig 2C, some transmission of the infection was only observed when the recipient mice were co-housed with index mice inoculated with the highest 10^9^ CFU dose immediately after inoculation. In this setting, 11 out of 15 recipient mice got stably infected by the index cage mates over the 7 days of co-housing and their nasal cavities harbored ~10^3^ to 10^5^ *B*. *pertussis* CFU on day 14 (Fig 2C). However, when initiation of co-housing of the inoculated index mice with recipient mice was delayed by 6 hours, only 2 out of 18 co-housed recipient mice got infected by *B*. *pertussis* transmission from inoculated index mice (Fig 2D). Collectively, these results show that even inoculation with very high numbers of *B*. *pertussis* bacteria did not allow to reproducibly overcome the colonization resistance of nasal mucosa of conventional C57BL/6J mice. Moreover, an extremely high inoculation and an immediate contact, likely enabling transfer of a portion of the applied inoculum to uninfected recipient mice upon physical contact, was required for transmission to occur. The human-adapted pathogen could only replicate up to a threshold of ≤10^6^ CFU per nasal cavity and was shed at a low level.

### Abrogation of TLR sensing enables *B*. *pertussis* proliferation on murine nasal mucosa and efficient transmission of bacteria between adult mice

**Fig 3:**
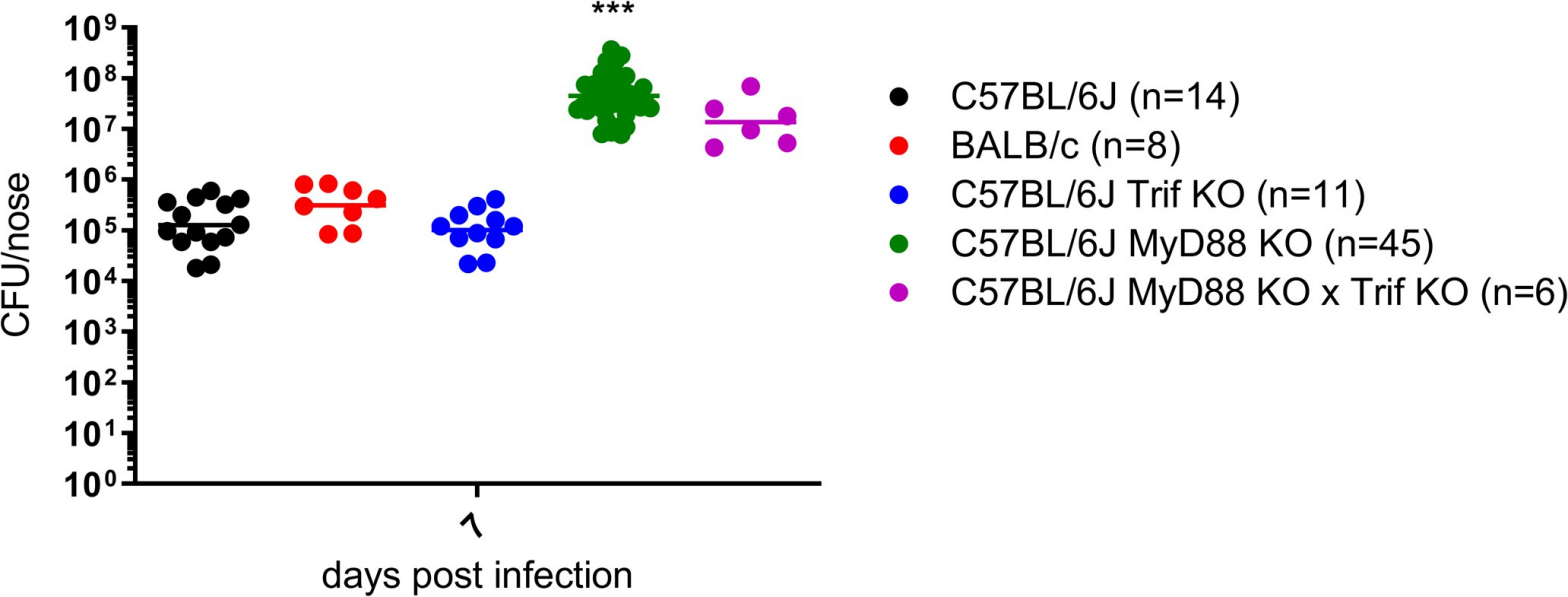
Ablation of MyD88-dependent TLR signaling enables efficient *B*. *pertussis* proliferation on murine nasal mucosa. Nares of immunocompetent inbred (BALB/c, C57BL/6J) and genetically defined immunocompromised (C57BL/6J MyD88 KO, C57BL/6J TRIF KO and C57BL/6J TRIF KO x MyD88 KO) mice were inoculated with 10^7^ CFU of *B*. *pertussis* suspended in 5 μl and seven days later the bacterial loads in their nasal cavities were determined as above. Dots represents bacterial counts of individual mice and horizontal bars represent the geometric means. Pools of values from several independent experiments are shown and the numbers of animals per tested mouse strain are given in brackets. Statistical significance of the CFU count difference between the C57BL/6J mouse group and other mouse groups was assessed by one-way ANOVA followed by Dunnett’s multiple comparison test. *** (p < 0.001).

The very high numbers of inoculated *B*. *pertussis* bacteria were expected to outcompete the resident microbiota components from epithelial adhesion sites, but only low level of *B*. *pertussis* colonization was observed 7 days after a high dose inoculation. This indicated that *B*. *pertussis* proliferation on the murine nasal mucosa was restricted by yet another mechanism than microbiota-conferred colonization resistance. We thus tested if the activation of innate immune defense mechanisms by pattern recognition receptors, sensing bacterial presence, limited *B*. *pertussis* proliferation on murine nasal mucosa. Since airway epithelial cells and the residing and/or infiltrating sentinel myeloïd phagocytes express Toll-like receptors (TLRs) as key sensors of bacterial cell components, we examined *B*. *pertussis* proliferation in the nasal cavity of TLR signaling-deficient mice. Infection experiments were performed using homozygous C57BL/6J-derived MyD88 (^-^/^-^) knock-out (MyD88 KO) mice deficient in signaling of all TLRs through the MyD88 adaptor and retaining only the TRIF-mediated endosomal TLR4 and TLR3 signaling pathways [38]. Further, homozygous TRIF KO mutant mice and a double MyD88 KO x TRIF KO mouse mutant deficient in all TLR-activated signaling mechanisms, were tested. Inoculation doses of 10^7^ CFU of *B*. *pertussis* cells were applied in 5 μl volumes into mouse nares and colonization of nasal cavities of the immunodeficient mice was compared to that of C57BL/6J and BALB/c immunocompetent inbred mice on day 7 after infection. As shown in Fig 3, while the bacterial counts did not exceed 10^6^ CFU in the nasal cavities of the C57BL/6J, BALB/c, or C57BL/6J-TRIF KO mice, *B*. *pertussis* was able to proliferate up to ~8 x 10^7^ CFU in the nasal cavity of the MyD88 KO mice, hence equally well as in the double MyD88 KO x TRIF KO mice. It can thus be concluded that rather than a limited number of bacterial adhesion sites, mouse-specific microbiota, or limited nutrient access, it was the signaling of TLR(s) through the MyD88 pathway and activation of downstream immune defense mechanisms that restricted *B*. *pertussis* proliferation on murine nasal mucosa.

**Fig 4:**
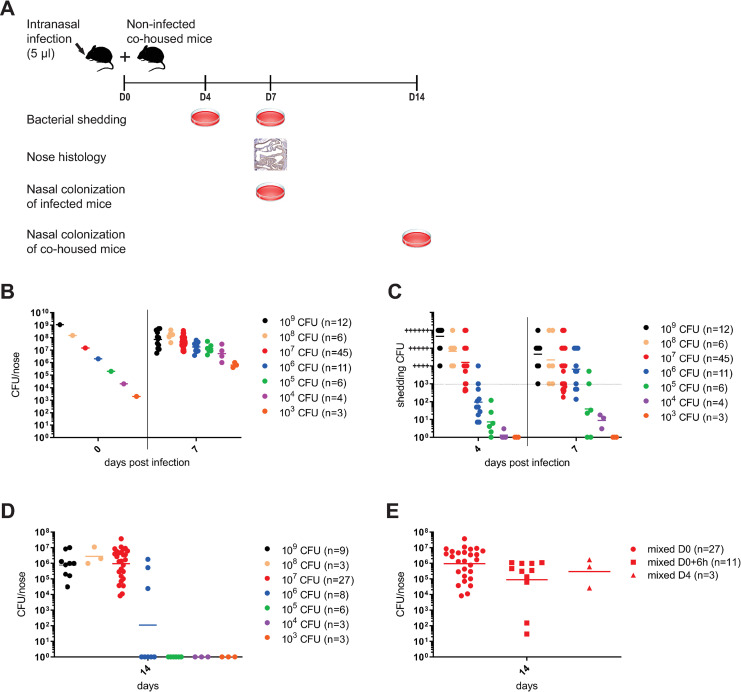
High bacterial proliferation in the nasal cavity triggers efficient nasal shedding and robust *B*. *pertussis* transmission between adult MyD88 KO mice. (**A**) Schematic depiction of the design of inoculation, nasal shedding, bacterial transmission and pathology assessment experiment. Nares of mice were inoculated with 5 μl of suspension containing indicated CFU content and inoculated index mice were placed into the same cage with non-inoculated recipient mice at a 1:1 ratio for co-housing. On days 4 and 7 nasal shedding of bacteria by index mice was determined by tapping their noses on BG plates and the index mice were next sacrificed for determination of bacterial loads in their nasal cavities and histological analysis of nasal cavity pathology. Co-housed recipient mice were kept for one week more and transmission-acquired *B*. *pertussis* loads in their nasal cavities were determined. (**B**) Nares of MyD88 KO mice were inoculated with 5 μl of bacterial suspensions containing from 10^3^ to 10^9^ CFU of *B*. *pertussis* cells and bacterial loads in infected nasal cavities were determined on day 7 by plating of nose homogenates. (**C**) Prior to euthanasia, *B*. *pertussis* shedding from the nasal cavities of infected mice was quantified on days 4 and 7 as described above. Shedding exceeding 10^3^ CFU per mouse nose/plate (dotted line) prevented more accurate CFU counting and its level was estimated from the density of the confluent *B*. *pertussis* lawns growing on BG agar and scored by the number of + signs indicating the estimated order of magnitude of shed bacterial numbers. (**D**) Index MyD88 KO mice inoculated into nares with the indicated dose of *B*. *pertussis* CFUs were co-housed in the same cage in a 1:1 ratio with non-inoculated recipient MyD88 KO mice for 7 days before the index mice were withdrawn for determination of nasal shedding and nasal cavity bacterial loads. The co-housed recipient mice were kept for another week prior to determination of bacterial loads in their nasal cavities on day 14. (**E**) In parallel, further index MyD88 KO mice inoculated with 10^7^ CFU of *B*. *pertussis* were kept in separate cages for 6 hours or 4 days before being brought in contact and co-housed in a 1:1 ratio over 7 days with the non-infected recipient mice and nasal shedding, nose colonization level and transmission to recipient mice was analyzed as above. Dots represent bacterial counts for individual mice. Horizontal bars indicate the geometric means. The data for mice inoculated with 10^7^ CFU were pooled for the 45 mice used in nine independent experiments (n = 45). The number of mice used per infection dose is given in brackets.

Therefore, a series of nose infection experiments was specifically designed using the MyD88 KO mice to assess the shedding of *B*. *pertussis* and explore a possible transmission of infection, as outlined in Fig 4A. Inoculation of nares of MyD88 KO mice by a series of bacterial doses spanning 6 orders of magnitude of CFU counts revealed that *B*. *pertussis* can proliferate from 10^3^ to 10^6^ CFU within 7 days in the nasal cavity of the MyD88 KO mice (Fig 4B). Further, upon inoculation of 10^8^ or 10^9^ CFUs the bacterial load after 7 days leveled at ~5 x 10^8^ CFU, indicating an upper limit of *B*. *pertussis* bacteria numbers accommodated in the nasal cavity of MyD88 KO mice. As further shown in Fig 4C, the high level of nasal cavity colonization translated into a sustained level of bacterial shedding by the infected MyD88 KO mice, as assessed on days 4 and 7 after inoculation. The MyD88 KO mice inoculated with the highest dose of 10^9^ CFU detectably shed *B*. *pertussis* for over 21 days (S1 Fig.). The level of bacterial shedding assessed on days 4 and 7 depended on the administered inoculation dose (Fig 4B). The lowest inoculation dose of 10^3^ CFU appeared insufficient to elicit detectable shedding of *B*. *pertussis* within 7 days from infection, whereas inoculation with 10^4^ or 10^5^ CFU already yielded substantial *B*. *pertussis* shedding on day 4, which increased by day 7 of infection. The mice inoculated with the highest doses of 10^7^ to 10^9^ CFU shed comparably high numbers of bacteria. These largely exceeded 10^3^ CFU per mouse nose, as judged from the density of the confluent *B*. *pertussis* lawns growing on BG agar and preventing more accurate CFU counting. Hence, with increasing bacterial load in the nasal cavity in time also the shedding of bacteria increased.

We next assessed the capacity of the infected MyD88 KO mice to transmit the infection to non-inoculated cage mates over 7 days of co-housing. As shown in Fig 4D, the rate of infection transmission was a function of the inoculation dose. Mice inoculated with 10^3^ to 10^5^ CFU failed to transmit the infection to co-housed non-inoculated mates, presumably due to the reduced levels of shedding developed over the 7 days after inoculation. Occasional transmission of infection was already observed with index mice inoculated with 10^6^ CFU, where three of the seven co-housed recipient mates got infected within 7 days and developed a colonization level of 10^4^ to 10^6^ CFU per nasal cavity on day 14 after in-cage exposure to index mice. Robust transmission of infection was then observed upon index mice inoculation with 10^7^ CFU. In this setting all 27 co-housed recipient MyD88 KO mice from several independent co-housing experiments got infected by *B*. *pertussis* transmission from index mice within 7 days of co-housing and by day 14 such recipient mice exhibited a bacterial load of 10^4^ to 10^7^ *B*. *pertussis* CFUs per nose. Moreover, the transmission was robust even if the recipient mice were added into co-housing cages 6 hours after index mice inoculation with 10^7^ CFU, where 11 out of 11 (100%) of recipient mice got infected as well (Fig 4E). Further, transmission also occurred when contact of recipient mice with inoculated index mice was delayed by 4 days from inoculation, where 3 out of 3 mice got infected by bacteria shed by the index mice. Hence, the MyD88 KO mice inoculated with 10^7^ CFU developed a sufficient level of colonization and shed *B*. *pertussis* efficiently enough to consistently transmit the infection onto co-housed recipient MyD88 KO cage mates. This inoculation dose was thus chosen for characterization of the pathology elicited in the nasal cavity of infected mice and for the subsequent infection transmission experiments with virulence factor mutants of *B*. *pertussis*.

### *Bordetella* infection causes inflammatory rhinosinusitis

**Fig 5:**
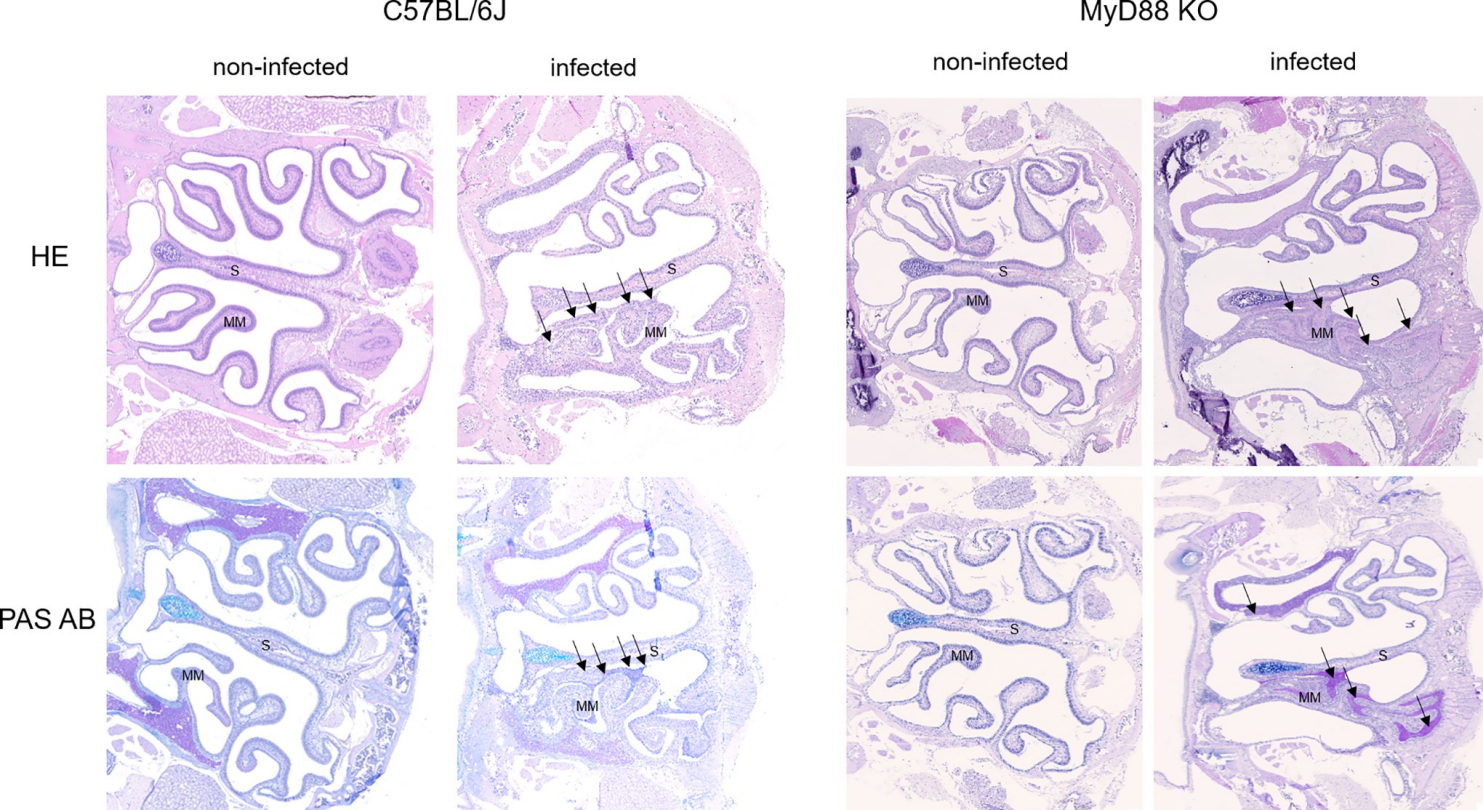
*B*. *pertussis*-induced infectious rhinitis pathology in the nasal cavity of MyD88 KO and C57BL/6J mice. Representative images of level 2 coronal sections through the nasal cavity of MyD88 KO and C57BL/6J mice on day 7 after inoculation of nares with 5 μl of suspension containing 10^7^ or 10^9^ CFU of *B*. *pertussis* bacteria, respectively. Compared to non-infected animals, bacterial colonization in the nasal cavities of infected mice (right panels) elicited mucus secretion and massive mucopurulent exudate accumulation in the cavities of the infected noses (black arrows) as revealed by hematoxylin and eosin staining (HE, upper panels) and Periodic Acid Schiff and Alcian Blue staining for mucus (PAS AB, lower panels). Septum (S), Middle Meatus (MM). Magnification 20x. 3 mice per group were used and representative images of stained sections are shown.

**Fig 6:**
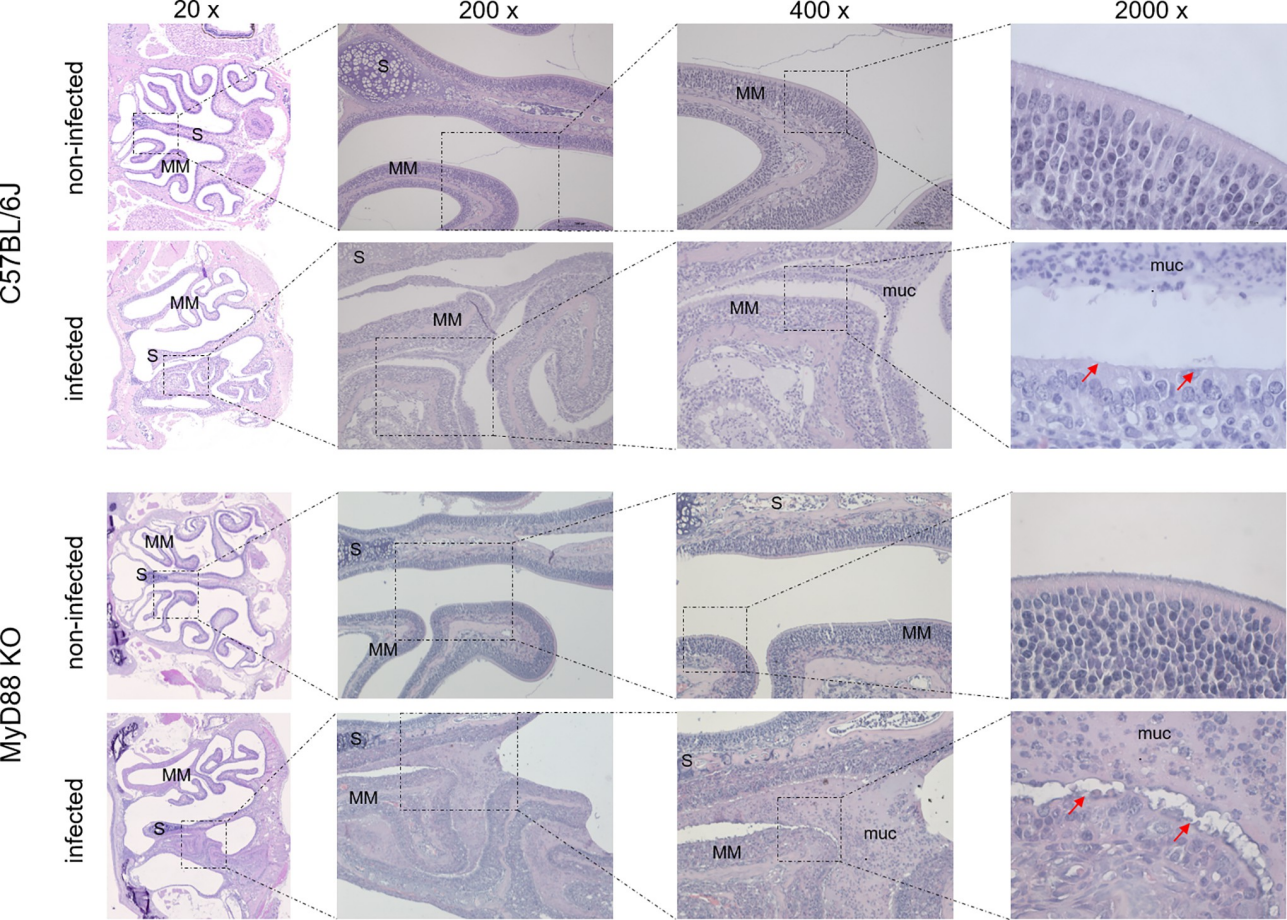
Details of *B*. *pertussis*-induced pathology in the nasal cavity of infected mice. Representative images of level 2 coronal sections through the nasal cavity of MyD88 KO and C57BL/6J mice on day 7 after inoculation of nares with 5 μl of suspension containing 10^7^ or 10^9^ CFU of *B*. *pertussis* bacteria, respectively. Zoom-out of HE-stained sections at magnifications up to 2000x. The red arrows indicate the denuded infected mucosa lacking the ciliated columnar epithelial cells (lower panel), as compared to healthy ciliated columnar epithelial layer observed on the middle meatus (MM) and nasal septum (S) in the nasal cavity of non-infected animals (upper panel). The olfactory epithelium and the surface of intranasal septum in infected mice is covered with a thick layer of mucus (muc.), the cavity is filled with mucopurulent exudate and the turbinates of the infected animals exhibit slight atrophy. 3 mice per group were used and representative images of stained sections are shown.

**Fig 7:**
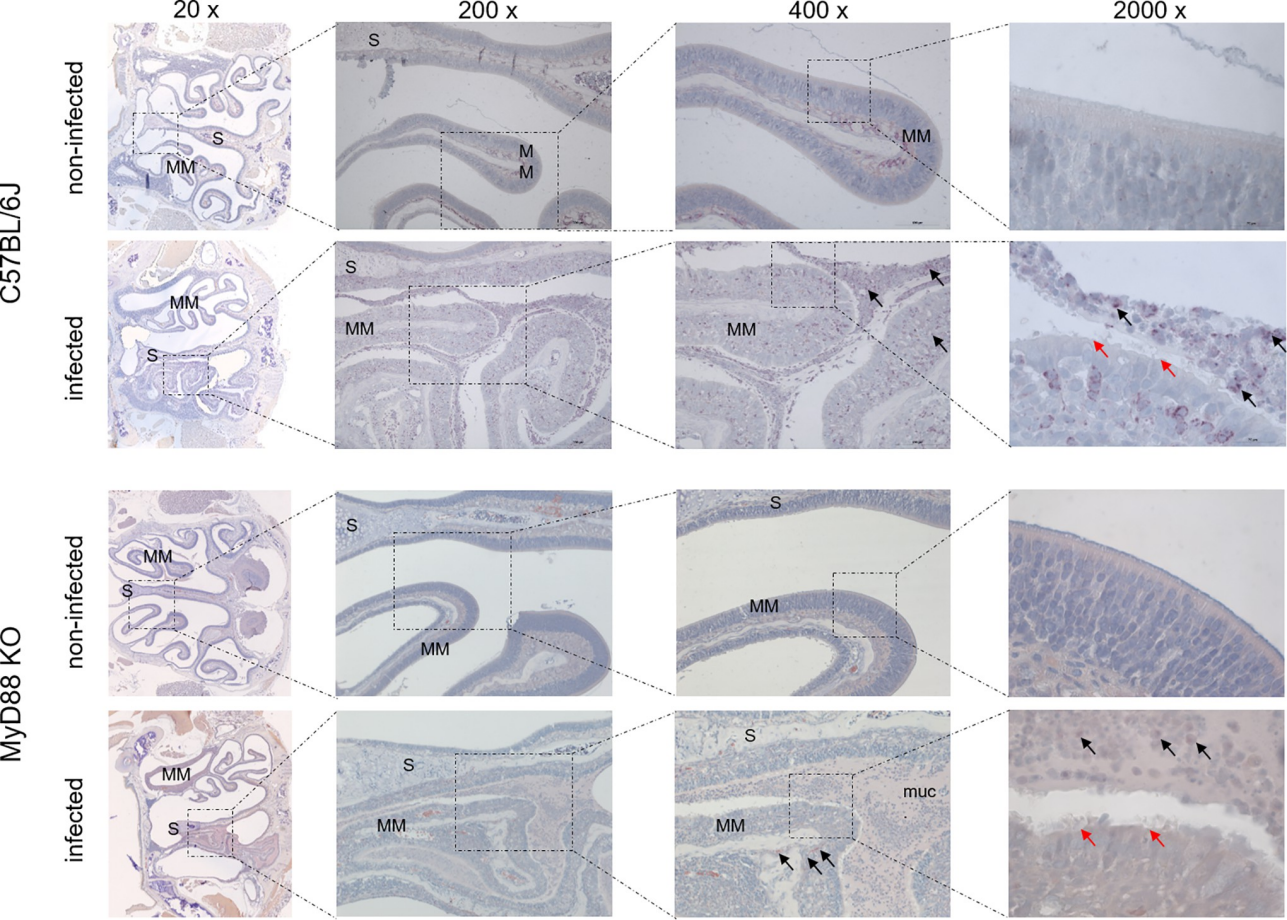
Neutrophil infiltration in the nasal cavity of *B*. *pertussis*-infected mice. NASDCL histochemistry detection of neutrophils. Representative images of NASDCL-stained level 2 coronal sections through the nasal cavity of MyD88 KO and C57BL/6J mice on day 7 after inoculation of nares with 5 μl of suspension containing 10^7^ or 10^9^ CFU of *B*. *pertussis* bacteria, respectively. The black arrows point to neutrophils present in high numbers in the mucopurulent exudate. The red arrow points to the disrupted layer of epithelium with loss of cilia. 3 mice per group were used and representative images of stained sections are shown.

**Fig 8:**
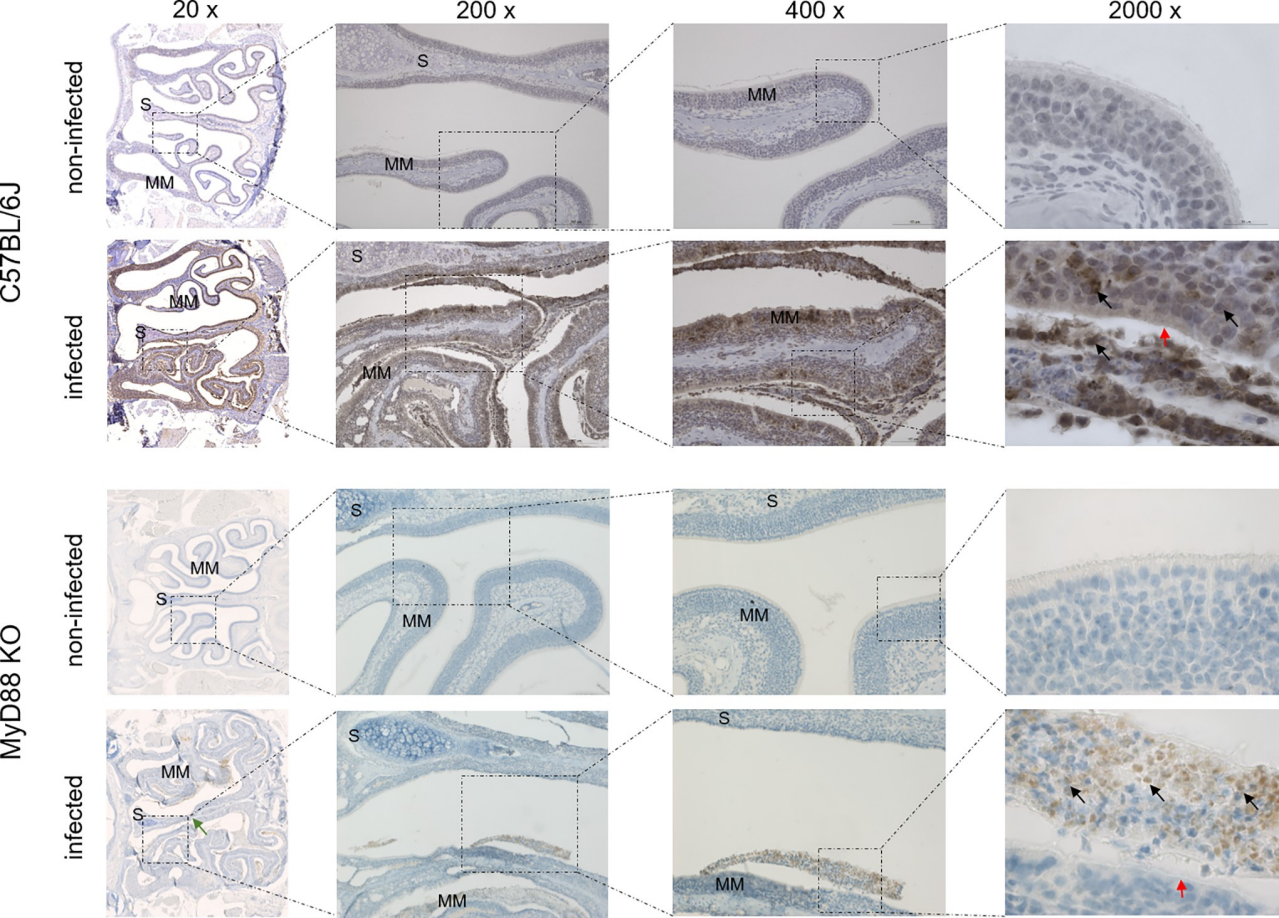
*B*. *pertussis* bacteria detection in the mucopurulent exudate filling the infected mouse nasal cavity. Immunohistochemical detection of *B*. *pertussis* bacteria. Representative images of stained level 2 coronal sections through the nasal cavity of MyD88 KO and C57BL/6J mice on day 7 after inoculation of nares with 5 μl of suspension containing 10^7^ or 10^9^ CFU of *B*. *pertussis* bacteria, respectively. Black arrows point to large amounts bacterial antigen present in the mucopurulent exudate, in part accumulating within phagocytic cell*s*. The green arrow points to atrophy of the septum. The red arrow points to the disrupted layer of epithelium with loss of cilia. 3 mice per group were used and representative images of stained sections are shown.

**Fig 9:**
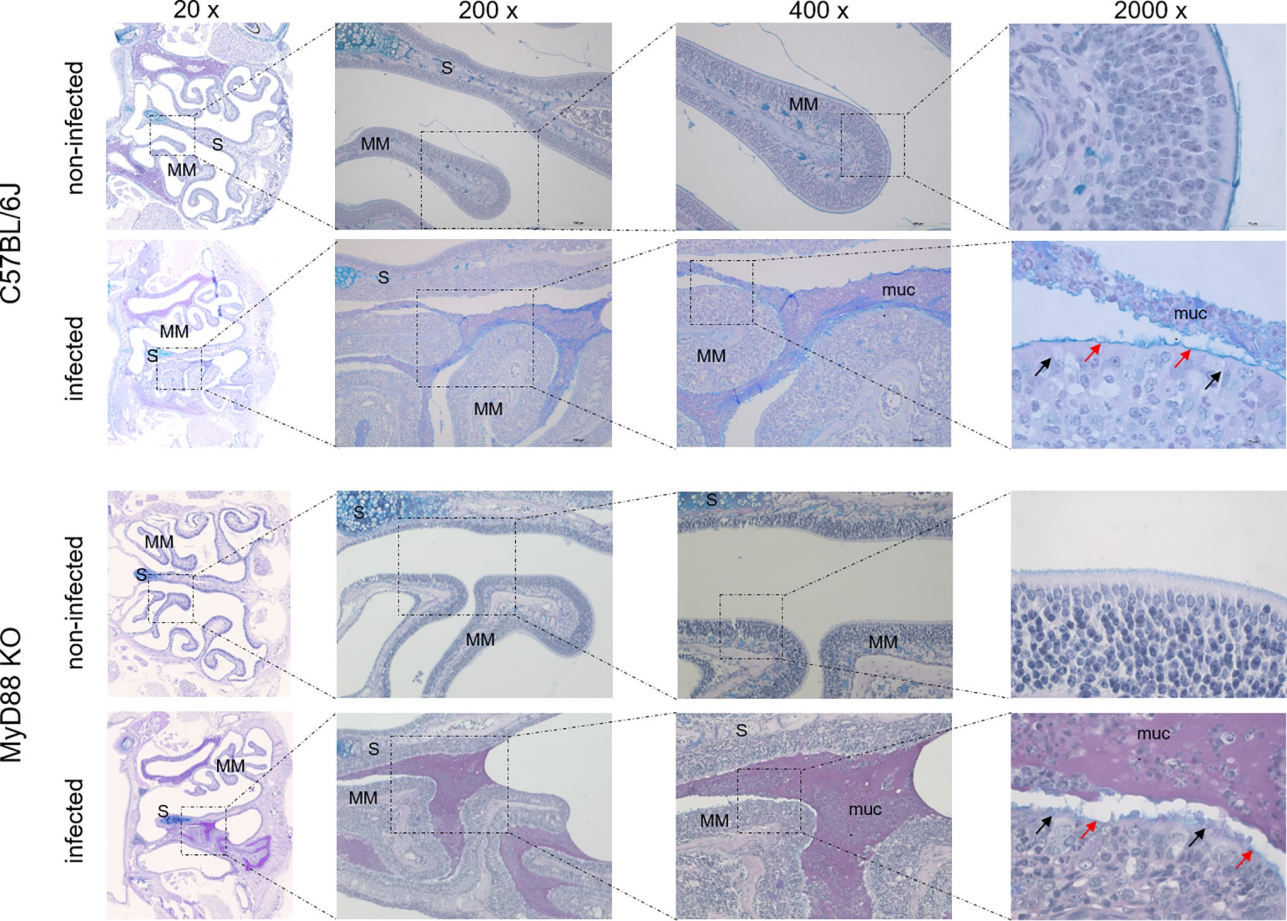
The *B*. *pertussis*-infected nasal cavity is filled by large amounts of mucinous exudate. Periodic Acid Schiff–Alcian Blue staining for mucus. Representative images of stained level 2 coronal sections through the nasal cavity of MyD88 KO and C57BL/6J mice on day 7 after inoculation of nares with 5 μl of suspension containing 10^7^ or 10^9^ CFU of *B*. *pertussis* bacteria, respectively. The olfactory epithelium and the nasal septum of infected mice is covered with a thick layer of mucus (muc) filling the cavities as mucopurulent exudate. Turbinates of the infected animals exhibit signs of atrophy. The red arrows point to the disrupted/denuded layer of olfactory epithelia that lost cilia. The black arrows point to the Goblet cells in the olfactory epithelium with massive mucus production. 3 mice per group were used and representative images of stained sections are shown.

**Fig 10:**
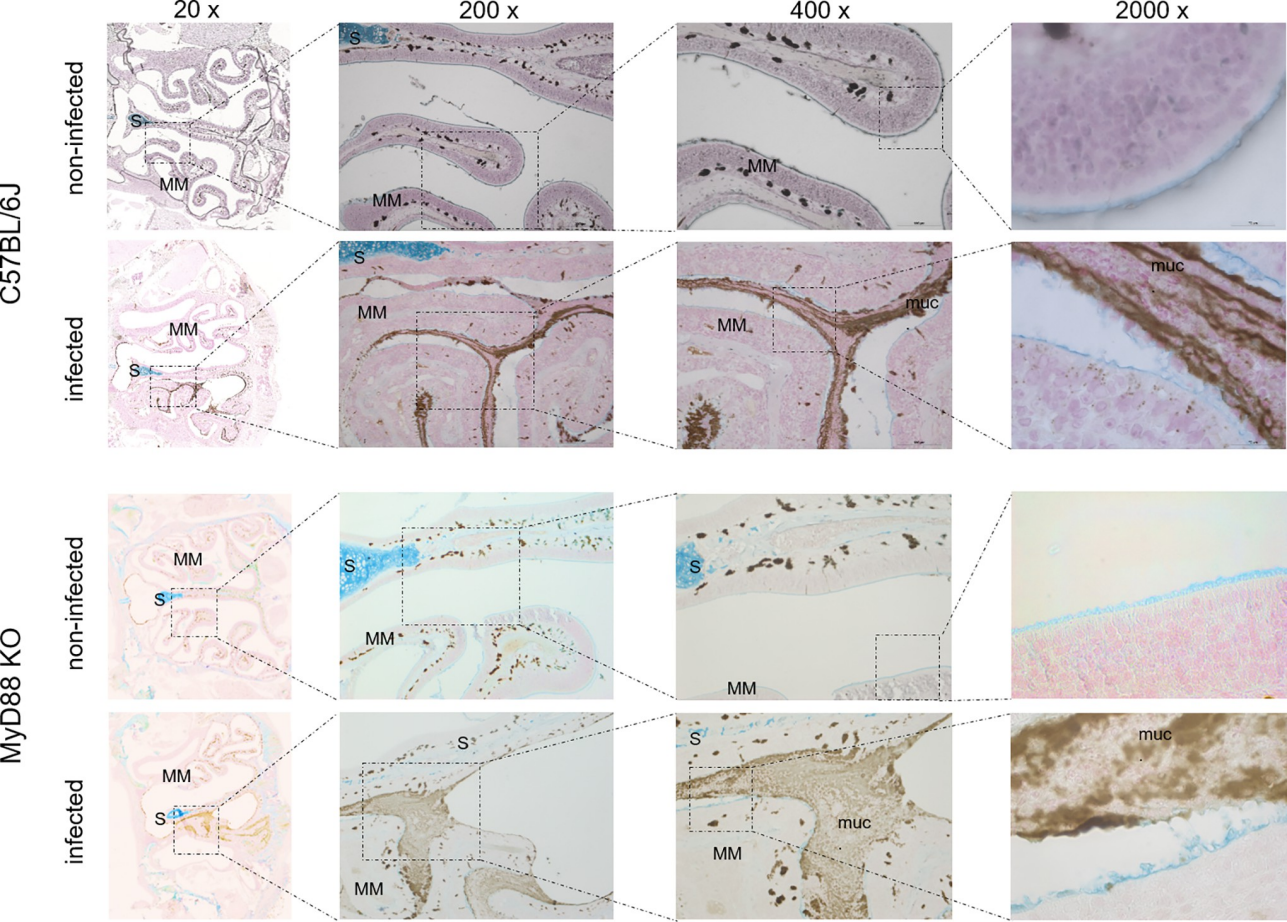
The mucinous exudate filling the *B*. *pertussis*-infected nasal cavity contains large amounts of Muc5Ac. Immunohistochemical staining for Muc5Ac. The mucopurulent exudate (muc) filling the cavities contains large amounts of Muc5Ac. Representative images of stained level 2 coronal sections through the nasal cavity of MyD88 KO and C57BL/6J mice on day 7 after inoculation of nares with 5 μl of suspension containing 10^7^ or 10^9^ CFU of *B*. *pertussis* bacteria, respectively. 3 mice per group were used and representative images of stained sections are shown.

**Fig 11:**
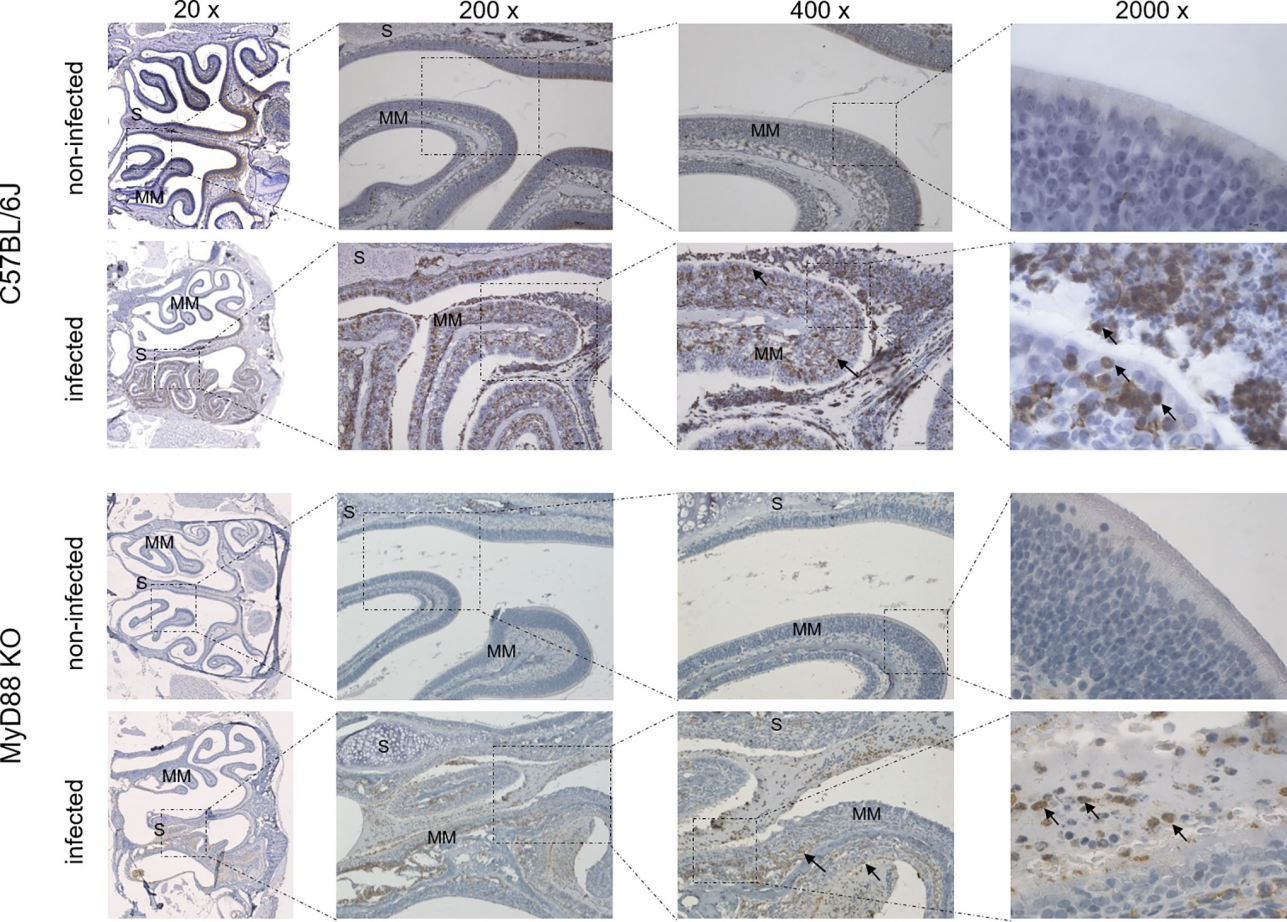
Macrophages are recruited into the *B*. *pertussis*-infected mouse nasal cavity. Immunohistochemical staining for the macrophage marker F4/80. Large numbers of macrophages (black arrows) are infiltrating the lamina propria and are detected in the mucopurulent exudate filling the cavities in infected mice. Representative images of stained level 2 coronal sections through the nasal cavity of MyD88 KO and C57BL/6J mice on day 7 after inoculation of nares with 5 μl of suspension containing 10^7^ or 10^9^ CFU of *B*. *pertussis* bacteria, respectively. 3 mice per group were used and representative images of stained sections are shown.

To investigate the upper airway pathology elicited by high level of *B*. *pertussis* proliferation in the nasal cavity, the MyD88 KO mice inoculated with 10^7^ CFU were sacrificed at the peak of infection on day 7, when also bacterial shedding was maximal. For comparison, the pathology in upper airways of C57BL/6J mice inoculated with a 100-fold higher dose of 10^9^ CFU was assessed as well and non-infected mice inoculated with the same volume of PBS were used as controls. Formalin fixed heads were decalcified and five coronal sections were paraffin-embedded and stained by several techniques for histopathological evaluation (Fig 5). Normal histology was observed for nasal sections from control animals inoculated by PBS only (Fig 5, left panels), exhibiting a clear nasal septum free of exudate and a healthy layer of pseudostratified columnar epithelium with well-preserved cilia. Representative images of nasal sections from both MyD88 KO and C57BL/6J mice infected with the two differing doses of *B*. *pertussis* (Fig 5, right panels) revealed a strongly increased mucus production with exudate eruption into the infected nasal cavities. Compared to healthy controls, severe nasal inflammation (rhinitis) with sinusitis was observed, extending throughout the length of the nose, with cavities filled with mucopurulent exudate (black arrows) that covered the focally denuded mucosa of the nasal cavity (Fig 6, 2000 x magnification). In the nasal cavities of infected animals the lumen between septum (S) and middle meatus (MM) was filled with thick layer of mucus and disruption of olfactory epithelium and loss of cilia was clearly apparent in both infected MyD88 KO and conventional C57BL/6J mice. The pathological changes were most pronounced in the distal part of the olfactory epithelium, on the septum and the middle meatus. The infected nasal mucosa exhibited clear signs of atrophy of ciliated epithelia of the nasal septum and turbinates, with extrusion of ciliated cells and a near-complete loss of ciliated columnar epithelia (Fig 6, 2000 x magnification, red arrows). Neutrophil infiltration (Fig 7, NASDCL staining 2000 x magnification, black arrows) and presence of necrotic changes of the structure of the epithelia and loss of goblet cells were apparent. The massive exudate within the cavities contained large numbers of bacteria (Fig 8, immunohistochemistry detection) and neutrophils (Fig 7, NASDCL staining), together with sloughed epithelial cells and necrotic debris embedded in basophilic mucinous exudate (Fig 8). Increased numbers of neutrophils obscured the underlying *lamina propria* (*cf*. Fig 7). Strongly enhanced mucus production was revealed by PAS AB staining (Fig 9), including massive Muc5AC excretion (Fig 10, immunohistochemistry). The maxillary turbinates were fused and the supporting bony structures were irregular and exhibited signs of atrophy (Fig 8, 20 x, green arrow). Overall, the NASDCL (Fig 7) and immunohistochemical F4/80 staining (Fig 11, 2000 x, black arrows) revealed massive infiltrations of neutrophils and macrophages into the infected mucosa and the mucinous exudate within the cavities. The observed pathology was quite comparable for the MyD88 KO mice and the C57BL/6J mice inoculated by a two orders of magnitude higher *B*. *pertussis* dose. These results clearly document that when delivered in a rather high dose into the nose, *B*. *pertussis* can induce massive infectious rhinitis in both conventional and the MyD88 KO mice, yielding significant inflammation, epithelial damage and mucus and exudate production.

### *B*. *pertussis* rhinitis eventually leads to lung infection

**Fig 12:**
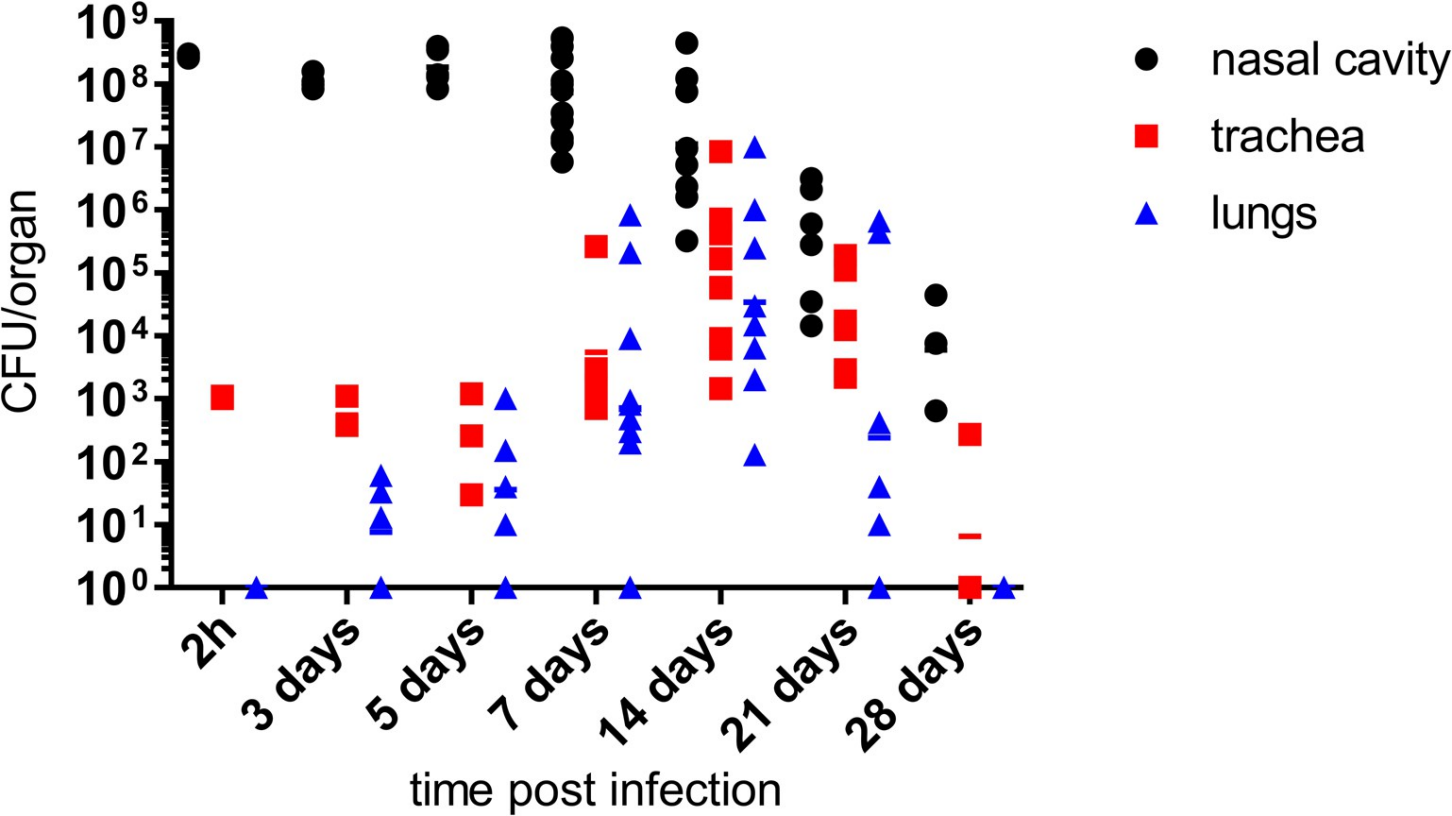
*B*. *pertussis* rhinosinusitis eventually leads to lung infection. Nares of MyD88 KO mice were inoculated with 10^9^ CFU of *B*. *pertussis* applied in 5 μl of suspension to prevent inhalation into lungs. At indicated time points the mice were sacrificed and bacterial loads in the nasal cavity, trachea and lungs were determined by plating of organ homogenates on BG agar. Three mice per time points of 2 h and 28 days were used and 5 to 12 mice were used for other time points. Data for days 7, 14 and 21 were pooled from two independent experiments and the lines correspond to geometric means.

The high level of infectious rhinitis, with accumulation of purulent mucus-containing exudate in the conchi of MyD88 KO mice in 7 days after high-dose inoculation, allowed to mimic the progressive evolution of the infection observed in human infants. About 5 days from inoculation of the nose with 10^9^ CFU, the initially low CFU counts in the trachea and lungs started to increase, indicating that infectious exudate from the nasal cavity reached the trachea and lungs and triggered a pneumonic infection that peaked at ~10^6^ CFU on day 14 after inoculation into nares (Fig 12). Beyond this time point the bacterial counts started to decrease both in the nasal cavity, as well as in the tracheas and lungs of infected mice, presumably due to onset of the adaptive immune response.

### Adhesins play a crucial role in colonization and transmission capacities of *B*. *pertussis*

*B*. *pertussis* is a particularly well-armed pathogen that produces two potent immunosuppressive protein toxins (PT, CyaA), as well as the Type III secretion system (T3SS)-excreted effector BteA/BopC and the dermonecrotic toxin (DNT), where the roles of BteA and DNT in *B*. *pertussis* virulence remain elusive [2,41]. The bacteria also produce an exopolysaccharide involved in *B*. *pertussis* biofilm formation on murine nasal septa [42] and *B*. *pertussis* acquires resistance to host antimicrobial peptides through an LgmB-catalyzed N-acetyl-glucosamine modification of lipid A phosphate groups of LOS [43]. The shed complement resistance protein Vag8 sequesters the C1q inhibitor and triggers futile complement consumption away from the bacterial surface [44]. Regulation of virulence genes expression is then fine-tuned by the RisA response regulator protein [45,46]. Further, *B*. *pertussis* sheds muramyl peptide fragments of peptidoglycan known to act as tracheal cytotoxin (TCT) that triggers NOD1 signaling and NO production, provoking damage of ciliated epithelial layers [47–49]. TCT shedding can then be reduced by introduction of a functional copy of the *ampG* gene into *B*. *pertussis* chromosome [50]. Finally, the pertactin (Prn), Fimbriae (Fim) and filamentous hemagglutinin (FhaB) adhesion factors were all shown to mediate attachment of *B*. *pertussis* to epithelial layers [2]. Moreover, Prn was also reported to mediate resistance to neutrophil-mediated clearance [51] and was recently implicated in triggering of shedding of the related *B*. *bronchiseptica* bacteria from the nasal cavity of mice [52]. Therefore, we constructed and tested an array of isogenic *B*. *pertussis* mutants, introducing a functional copy of the *ampG* gene, deleting virulence factor genes and/or introducing toxin activity-ablating point mutations onto genes on the bacterial chromosome, individually or in triplicate, quadruplicate or pentaplicate combinations, as specified in detail in Table 1. Using this mutant collection, we examined which of the known virulence factors were involved in the capacity of *B*. *pertussis* to colonize the nasal mucosa of the MyD88 KO mice and enabled it to shed and transmit in the newly established murine model of catarrhal *B*. *pertussis* infection.

**Table 1:** List of bacterial strains used in this study. Bacterial strain name, genotype description and reference are indicated.

*Bordetella pertussis* strain	Genotype and relevant description	Reference
WT	*Bp* CIP 81.32 WT; wild-type *Bordetella pertussis* Tohama I (CIP 81.32); *fim2-1*, *fim3-1*, *ptxP1*, *ptxA2*, *ptxB1*, *ptxC1*, *ptxD1*, *ptxE1*, *prn1*	[64,95]
*ΔbscN*	*Bp* CIP 81.32 with *bsc*N in-frame deletion of codons R2—E443	[41]
*Δbp*s	*Bp* CIP 81.32 with whole *bps* locus in-frame deletion of codons N2 of Bp1944*—*L196 of Bp1941	this study
*Δdnt*	*Bp* CIP 81.32 with *dnt* in-frame deletion of codons K3—P1463	this study
*Δvag8*	*Bp* CIP 81.32 with *vag8* in-frame deletion of codons A2—W915	this study
*ΔlgmB*	*Bp* CIP 81.32 with *lgm*B in-frame deletion of codons L3—G539	[43]
*ΔcyaA*	*Bp* CIP 81.32 with *cyaA* in-frame deletion of codons Q2—R1706	this study
*cyaA-AC* ^*-*^	*Bp* CIP 81.32 with insertion of codons GS between position 188 and 189 of the *cya*A gene	[29,96]
RisA D60N	*Bp* CIP 81.32 with point mutation in the codons D60N of the *ris*A gene	[46]
ΔTCT	*Bp* CIP 81.32 with insertion of *amp*G gene from *E*. *coli* to the *B*. *pertussis* pseudogenes Bp1271—Bp1272	this study
*Δprn*	*Bp* CIP 81.32 with *prn* in-frame deletion of codons N2—W910	this study
*Δptx*	*Bp* CIP 81.32 with whole *ptx* S1-S3 operon in-frame deletion of codons S1 R2—S3 C227	this study
*ptx* R9K E129G	*Bp* CIP 81.32 with double point mutation in the codons R9K and E129G of the *ptx* S1 gene	[29,97]
*Δfim*	*Bp* CIP 81.32 with whole *fim* operon in-frame deletion of codons I1 of *fim*A—N363 of *fim*D	this study
*ΔfhaB*	*Bp* CIP 81.32 with *fhaB* in-frame deletion of codons N4—T3588	this study
*ΔfhaB-fim*	*Bp* CIP 81.32 with *fhaB-fim* in-frame deletion of codons N4 of *fha*B—T361 of *fim*D	this study
3M	*Bp* CIP 81.32 with 3 mutations; *ptx* R9K E129G, *ΔlgmB*, *Δdnt*	this study
4M	*Bp* CIP 81.32 with 4 mutations; *ptx* R9K E129G, *ΔlgmB*, *Δdnt*, *cyaA-AC*^*-*^	this study
5M	*Bp* CIP 81.32 with 5 mutations; *ptx* R9K E129G, *ΔlgmB*, *Δdnt*, *cyaA-AC*^*-*^, *ΔbscN*	this study

**Fig 13:**
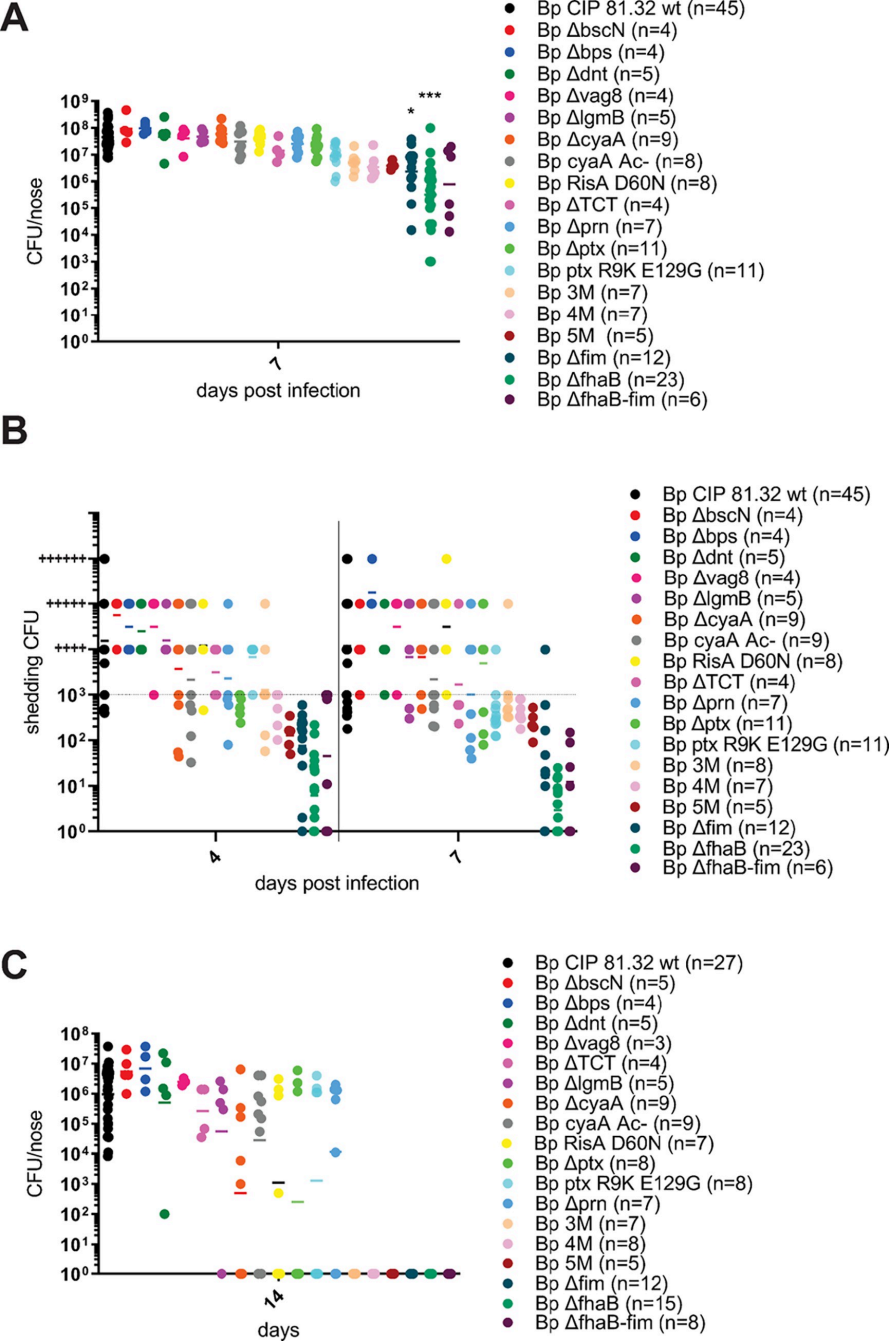
The FhaB and FIM adhesins play a crucial role in nasal cavity colonization and of *B*. *pertussis* transmission. (**A**) Nares of MyD88 KO mice were inoculated with 10^7^ CFU of the indicated *B*. *pertussis* mutant (Table 1) suspended in 5 μl and bacterial loads in infected nasal cavities were determined on day 7 by plating of nose homogenates. Statistical significance of CFU count difference between mice inoculated with the wild-type *B*. *pertussis* and groups of mice infected by the mutant strains was assessed by one-way ANOVA followed by Dunnett’s multiple comparison test. * (p < 0.05); *** (p < 0.001). (**B**) Prior to euthanasia, *B*. *pertussis* shedding from the nasal cavities of infected mice was quantified on days 4 and 7 as in Fig 4B. (**C**) Index MyD88 KO mice inoculated into nares with 10^7^ CFU of the indicated *B*. *pertussis* mutant were co-housed in the same cage in a 1:1 ratio with non-inoculated recipient MyD88 KO mice for 7 days before the index mice were withdrawn for determination of nasal shedding and nasal cavity bacterial loads. The co-housed recipient mice were kept for another week prior to determination of bacterial loads in their nasal cavities on day 14. Dots represent bacterial counts for individual mice. Horizontal bars indicate the geometric means. Several independent infection experiments were performed with *B*. *pertussis* mutants exhibiting reduced colonization, shedding and transmission capacities and the obtained CFU data were pooled. Mice inoculated with 10^7^ CFU of wild-type *B*. *pertussis* (45 in total) were used as internal standard in all experiments with *B*. *pertussis* mutants. The number of mice used per challenge strain is given in brackets.

**Fig 14:**
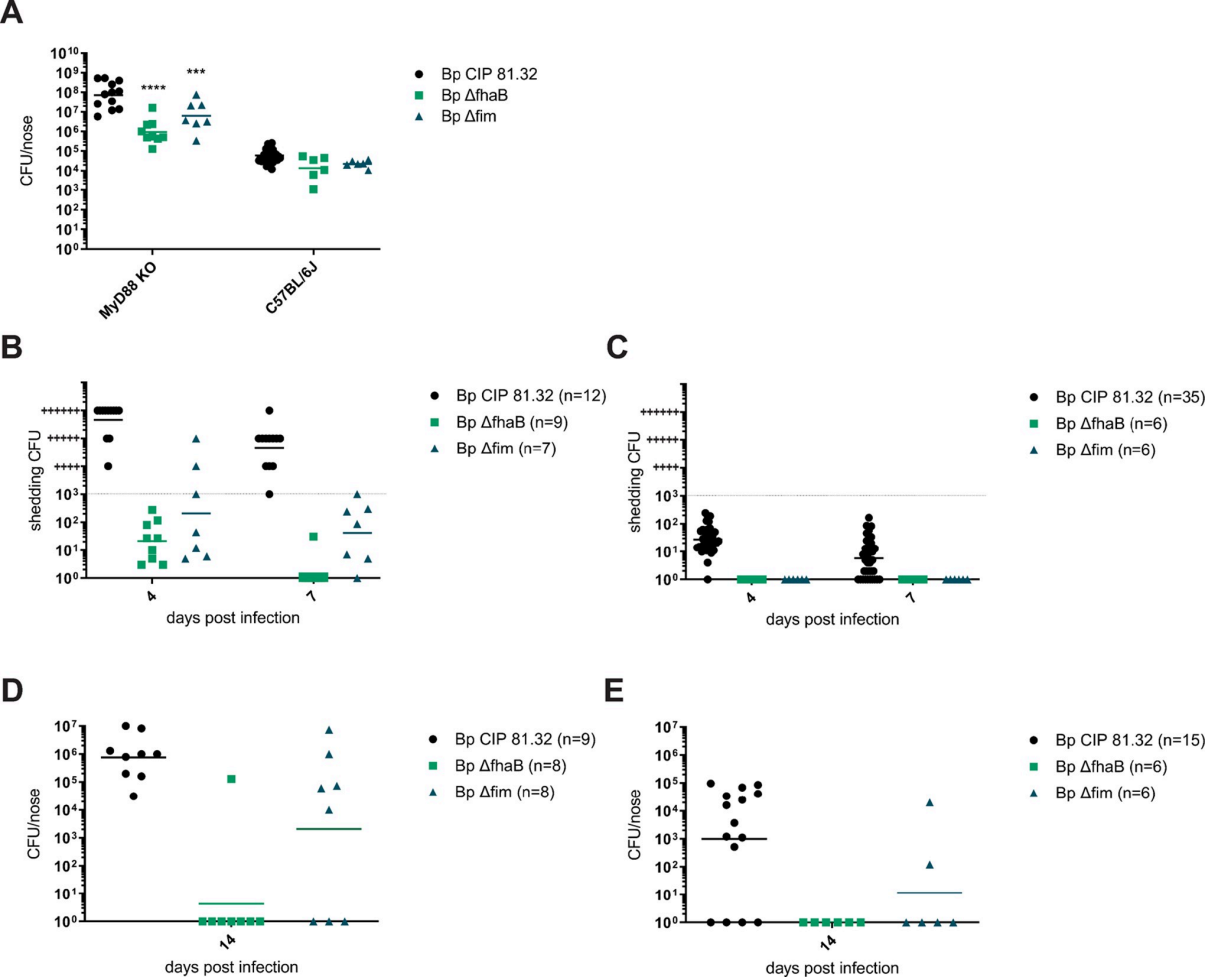
FhaB plays a key role in nasal cavity colonization and *B*. *pertussis* transmission. Nares of MyD88 KO and C57BL/6J mice were inoculated with 10^9^ CFU of the WT or the indicated *B*. *pertussis* mutants (*ΔfhaB* o*r Δfim)* supended in 5 μl. (**A**) Bacterial loads in the infected nasal cavities were determined on day 7 by plating of nose homogenates. Statistical significance of CFU count difference between mice inoculated with the wild-type *B*. *pertussis* and groups of mice infected by the mutant strains was assessed by one-way ANOVA followed by Dunnett’s multiple comparison test *** (p < 0.001), **** (p < 0.0001). (**B**) *B*. *pertussis* shedding from the nasal cavities of infected index MyD88 KO mice and (**C**) of index C57BL/6J mice was quantified on days 4 and 7 as described for Fig 4C. (**D**) Index MyD88 KO or (**E**) C57BL/6J mice inoculated into nares with 10^9^ CFU of the indicated *B*. *pertussis* mutant were co-housed in the same cage in a 1:1 ratio with non-inoculated recipient MyD88 KO or C57BL/6J mice for 7 days before the index mice were withdrawn for determination of nasal shedding and nasal cavity bacterial loads (cf. B and C). The co-housed recipient mice were kept for another week prior to determination of bacterial loads in their nasal cavities on day 14. Dots represent bacterial counts in nasal cavities of individual recipient MyD88 KO (**D)** and C57BL/6J (**E**) mice, respectively. Horizontal bars indicate the geometric means. The number of mice used per challenge strain is given in brackets.

As summarized in Fig 13A, 10^7^ CFU doses of various mutant strains were inoculated in 5 μl suspension volumes into mouse nares and nasal cavity colonization levels were assessed in nose homogenates on day 7 after infection. Quite unexpectedly, a broad variety of mutants did not exhibit any major defect of nasal cavity colonization capacity. A mildly reduced colonization was observed for the bacteria secreting a genetic toxoid of PT (PtxS1-R9K/E129G) alone, or in combination with deletions of the *lgmB* and *dnt* genes (3M), or when the CyaA toxin was genetically detoxified (4M) as well, and/ or when also the deletion of the T3SS apparatus ATPase *bscN* gene was added (5M), respectively. Intriguingly, the defect of colonization capacity caused by elimination of the immunosuppressive enzymatic activities of the PT and CyaA toxins was rather modest in the used immunocompromised mouse infection model. Similarly, defects in TCT shedding (*E*. *coli ampG* gene knock-in), DNT (Δ*dnt*), LgmB (Δ*lgmB*) and PRN (Δ*prn*) protein production, RisA activity (D60N substitution), exopolysaccharide production (Δ*bps*), complement resistance (Δ*vag8*) or BteA excretion by the T3SS (Δ*bscN*) had little impact. In contrast, a significant decrease of nasal cavity colonization capacity was observed for bacteria lacking the fimbriae due to deletion of the entire *fim* operon (Δ*fim*), or lacking the filamentous hemagglutinin protein precursor (FhaB) due to deletion of the *fhaB* gene (Δ*fhaB*), or lacking both adhesins (*ΔfhaB-fim*). Compared to the other mutants, the Δ*fhaB* and Δ*fim* mutants reached about an order of magnitude lower counts in the nasal cavity and exhibited a bi-modal behavior, with the CFU counts recovered from the noses of some mice being several orders of magnitude lower than from other mice that received the same inoculation dose. Moreover, the Fim and FhaB-deficient mutants were shed on days 4 and 7 after inoculation at significantly lower levels from the nasal cavity of infected mice than the other examined mutants (Fig 13B). Finally, alike the 3M, 4M and 5M mutants deficient in production of several toxins and virulence factors at a time, the Δ*fim* and Δ*fhaB* mutants failed to transmit from infected index mice to co-housed MyD88 KO mice, as judged from the failure to establish a productive infection in nasal cavities of co-housed mice (Fig 13C). Moreover, the colonization, shedding and transmission defects could not be alleviated by a 100-fold increase of the inoculation dose to 10^9^ CFU (Fig 14). A particularly strong phenotype was observed for the Δ*fhaB* mutant, which colonized the nasal cavity at about ten-fold lower levels than the Δ*fim* mutant (Fig 14A), was shed at about ten-fold lower levels (Fig 14B) and essentially failed to transmit, with only 1 out of 8 co-housed recipient MyD88 KO mice being productively infected to a detectable level, compared to 5 out of 8 co-housed mice infected by the Δ*fim* mutant (Fig 14D). Importantly, no shedding or transmission of the Δ*fhaB* mutant was observed in the immunocompetent C57BL/6J mice despite the very high inoculation dose (Fig 14C and 14E), whereas 2 out of 6 co-housed mice got infected by the Δ*fim* mutant (Fig 14E). The fimbriae and in particular the FhaB protein thus represent key factors required for proliferation of *B*. *pertussis* in the murine nasal cavity to levels that can elicit bacterial shedding and productive transmission to new hosts.

## Discussion

We used immunocompromised MyD88 (^-^/^-^) knock-out mice to model the catarrhal phase of *B*. *pertussis* infection and report for the first time that robust transmission of the human-adapted pathogen can occur in adult mice. Screening of a set of *B*. *pertussis* mutants for transmission factors in this murine model revealed the crucial role of the FhaB and Fim adhesins in colonization of the nasal cavity, shedding and transmission of *B*. *pertussis*.

Using a very high inoculation dose of 10^9^ CFU, a catarrhal rhinosinusitis pathology could be also induced in the nasal cavity of the immunocompetent parental C57BL/6J mice and its manifestations were similar to that induced in the nasal cavity of the immunocompromised MyD88 KO mice inoculated with a 100-fold lower bacterial dose. Indeed, some shedding and transmission of *B*. *pertussis* was observed also with the highly inoculated C57BL/6J mice and the requirement for FhaB and Fim for bacterial shedding and transmission could be validated also in the immunocompetent mice (*cf*. Fig 14). However, despite the extremely high inoculation dose, transmission of *B*. *pertussis* between immunocompetent C57BL/6J mice was not robust and required that the inoculated index mice were brought in contact with recipient mice very shortly after inoculation (*cf*. Fig 2D). This was likely due to rapid reduction of the number of viable bacteria in the nasal cavity of C57BL/6J mice following upregulation of a vigorous antimicrobial peptide response within 24 hours after infection. Indeed, the C57BL/6J mice resisted infection even when co-housed with the MyD88 KO mice inoculated by 10^7^ CFU, which shed rather high numbers of *B*. *pertussis* bacteria (S2 Fig).

An obvious limitation of the developed MyD88 KO mouse model of catarrhal pertussis consists in the artificial reduction of *B*. *pertussis*-host interaction complexity through ablation of the key TLR signaling pathways that activate innate and adaptive immune responses to bacterial infection. The MyD88-dependent TLR2 and TLR4 signaling of the presence of bacterial lipoprotein/lipopeptide and lipooligosaccharide was, indeed, shown to play an important role in mobilization and orchestration of the innate immune response of airway mucosa to *B*. *pertussis* infection [40,53–58]. Nevertheless, a massive rhinosinusitis and catarrh accompanied by accumulation of mucopurulent exudate still developed in the nasal cavity of the immunodeficient MyD88 KO mice at the peak of infection, once a human-like high bacterial colonization level was reached. Hence, the proinflammatory signaling of the remaining MyD88-independent mechanisms of multi-receptor sensing of bacterial molecular patterns [59] still ensured massive phagocyte chemoattraction to the infected nasal mucosa. Together with sensing of epithelial and innate immune cell injury resulting from the action of *B*. *pertussis* toxins (e.g. CyaA, PT, DNT, TCT) [36,47–49,60–65], such MyD88-independent danger signaling enabled the progressive resolution of the infection at later time points, beyond week four of infection, when the activated adaptive immune responses come in play (*cf*. Fig 12). The MyD88 KO mouse model can thus still be plausibly used for dissection of host molecular mechanisms that are hijacked by bacterial transmission factors to ensure high *B*. *pertussis* transmission rates. The caveat to be kept in mind is that most *B*. *pertussis*-infected humans are immunocompetent, albeit the innate immune system of infants is notorious for its immaturity and is suppressed by persisting erythroid TER119^+^ CD71^+^ cells [66,67]. In this respect the MyD88 KO mice model may mimic to some extent the poor innate immune defense of infant mucosa that needs to remain permissive for colonization by useful microbiota early in life. In an immunocompetent host the synergy of immunosuppressive actions of PT and CyaA on myeloïd and epithelial cells in concert with other virulence factors will dampen the innate immune response and enable the observed high-level proliferation of *B*. *pertussis* on nasopharyngeal mucosa. It should further be noted that there exist important anatomical and physiological differences in the nasal cavity architecture, submucosal gland localization, respiratory epithelial cell type distribution and neurophysiological regulation of airway mucus production between mice and humans [68,69]. The volume of the human nasal cavity is about 800-fold larger (25 cm^3^ versus 0.03 cm^3^) and its surface is about 55-times larger (60 cm^2^ versus 2.89 cm^2^) than that of mice [70]. Given that in the undiluted nasal aspirates of infants the *B*. *pertussis* densities can be approaching ~10^8^ CFU/ml [3], it is plausible to assume that the real numbers of *B*. *pertussis* bacteria present in the infected nasal cavities of infants may still be one or two orders higher, approaching 10^9^ to 10^10^ CFU forming a biofilm on ciliated nasal epithelia [42]. In this respect the ~5 x 10^7^ to 10^8^ CFU *B*. *pertussis* counts reached in the nasal cavity of MyD88 mice may proportionally well mimic the extent of bacterial colonization of human nasal cavity during catarrhal pertussis.

Until now, *B*. *pertussis* transmission was only achieved in the neonatal mouse model [36] that bears technical constraints limiting the robustness of *B*. *pertussis* transmission studies. A model reproducing more robustly the human pathology and immune response has previously been established using newborn piglets infected at 1 to 4 weeks of age [71,72]. The clinical symptoms of catarrhal infection accompanied by nasal discharge and non-paroxysmal cough could be reproduced in newborn piglets, making it likely that *B*. *pertussis* transmission studies could be robustly performed in the piglet model as well. Regrettably, the piglet model has so far been used by a single team, presumably because of high operational costs and limited access to specialized facilities for infectious challenge experiments in piglets. Moreover, targeted inbred pig mutant lines for mechanistic dissection of the studied pathophysiological phenomena remain unavailable. Recently, the full clinical course of human pertussis with development of characteristic disease symptoms, including aerosol transmission of the infection to naïve animals, was reproduced in olive baboons [34,35,73,74]. While excelling in the approximation of human pertussis pathophysiology, this model of outbred non-human primates remains limiting in terms of associated costs, low numbers of available animals, complex ethical considerations and the impossibility of targeted mutagenesis of primates for genetic dissection of the studied mechanisms. Interestingly, a genetically tractable pertussis model of coughing rats was established and used for vaccine testing some 3 decades ago [75,76]. Recently, this model was brought to a higher technical level by application of whole body plethysmography on Sprague-Dawley rats [77]. Since rats are nowadays amenable to targeted genetic modification, the development of specific rat mutants for deciphering of molecular mechanisms of early postnasal drip elicited cough and subsequent development of the paroxysmal cough syndrome of *B*. *pertussis* infection remains an attractive option for future studies. In the light of the above, the here-established MyD88 KO mouse model of catarrhal disease and *B*. *pertussis* transmission offers the advantage of accessible cost and of numerous readily accessible facilities for SPF mice breeding and infection experiments in mice in all major infection research centers. The particular attraction of the mouse model then consists in the power of the highly developed genetic manipulation technology, with already available comprehensive archives of mouse mutants and the nowadays routinely used technologies for targeted mutagenesis of mice available also on commercial basis. This offers unparalleled opportunities for identification and dissection of host molecular mechanisms that are hijacked by bacterial factors for the purpose of *B*. *pertussis* transmission. In combination with the straightforward genetic manipulation of the pathogen, this offers an unprecedented option for mechanistic studies on catarrhal pertussis disease development and identification of the involved bacterial factors to be targeted by future vaccination strategies.

The choice of immunodeficient MyD88 KO mouse as a model for nasal shedding and transmission of *B*. *pertussis* was in part guided by the previous observation that TLR4 signaling limits the transmission of the rodent pathogen *B*. *bronchiseptica* in mice [78]. Expectedly, a functional TLR4 sensor was previously found to be required for an early TNF-α, IL-1β and IFN-γ response of mice to *B*. *pertussis* infection and enhanced clearance of the bacteria [53]. Indeed, some extent of *B*. *pertussis* transmission and rhinitis could recently also be observed in infection experiments performed (with our involvement) in TLR4-deficient C3H/HeJ mice by the collaborating team of Dr. E.T. Harvill at University of Georgia [79]. Not surprisingly then, we found that blunting of all but endosomal TRIF-mediated signaling of the key TLR sensors of epithelial cells and phagocytes in the MyD88 KO mice allowed the human-adapted *B*. *pertussis* to breach the colonization resistance of murine nasal mucosa. The pathogen proliferated within the murine nasal cavity to numbers approaching those found in nasal aspirates of diseased human infants [3] and the heavily *B*. *pertussis*-infected nasal mucosa exhibited typical signs of infectious rhinitis. This was accompanied by massive mucus and mucopurulent exudate accumulation in the nasal cavity, loss of columnar ciliated epithelial cell layers, signs of turbinate atrophy, massive bacterial shedding and efficient *B*. *pertussis* transmission to co-housed naïve MyD88 KO mice. This would fairly well reproduce most of the catarrhal and early paroxysmal phase manifestations of pertussis disease in humans. It will be of interest to use the specialized equipment that has recently made possible to assess whether the infected mice also develop sneezing and postnasal drip-elicited cough [80,81]. Consequently, *B*. *pertussis* aerosolization by infected mice was not monitored here and remains to be explored in future studies.

Infectious nasal discharge is readily induced in humans by various agents, while it is nontrivial to observe in mice. We assume that the method of tapping infected mouse nose tips on BG agar allowed a semiquantitative assessment of the nasal discharge that resulted from *B*. *pertussis* colonization of mouse nasal cavity, although visual inspection did not unambiguously indicate the occurrence of nasal discharge in infected mice. Worth of note, MyD88 deficiency was reported to yield mucous cell metaplasia and upregulation of mucus production, with enhanced mucocilliary clearance, under conditions of high level exposure to bacterial components, compensating in part for reduced proinflammatory signaling in the absence of MyD88 [82]. It will be of interest to explore to which extent the enhanced mucus production contributed to the robustness of bacterial shedding and transmission in the MyD88 KO mice and whether the loss of the key TLR signaling paths was also compensated for by enhanced plasma leakage from *lamina propria* onto the mucosal surface in the MyD88 KO mice. Here we defined the infectious dose and experimental conditions, under which consistent shedding and transmission of *B*. *pertussis* occurred in the MyD88 KO mice. This opens the way to a detailed comparative analysis of cellular compositions, cytokine and antimicrobial peptide production and gene expression profiles of the infected epithelial layers and NALT tissues of immunocompetent and MyD88 KO mice. Such experiments are currently underway and are expected to yield hints on the involved host mechanisms that are triggered during catarrhal shedding and transmission phase of *B*. *pertussis* infection.

On the side of the pathogen the here-presented mouse model already allowed to examine the involvement of a number of virulence factors in transmission of *B*. *pertussis*. In line with observations of Scanlon and coworkers in the neonatal mouse model [36], the absence of the pertussis toxin (*Δptx*) did not affect the nasal cavity colonization and *B*. *pertussis* transmission capacity any importantly. In line with that, we observed that genetic inactivation of the key immunosuppressive CyaA and PT toxins had only a minor impact on the capacity of such toxin-deficient *B*. *pertussis* mutants to colonize the nasal mucosa of immunodeficient MyD88 KO mice. These toxins are required for efficient airway colonization in immunocompetent mice particularly in the early phases of infection, where the CyaA and PT toxin activities interfere with the TLR signaling-activated chemoattraction and bactericidal activity of sentinel phagocytes, inhibiting also the production of antimicrobial peptides by epithelial cells and enabling bacterial proliferation on the nasopharyngeal mucosa [60,62,83,84]. In the MyD88 KO mice deficient in TLR signaling the mobilization of innate bactericidal defenses will likely be delayed and thus the importance of CyaA and PT action for nasopharynx colonization by *B*. *pertussis* may plausibly be expected to be of reduced importance. Along the same line, many of the here examined *B*. *pertussis* mutants defective in the mechanisms conferring antimicrobial peptide resistance (*ΔlgmB*), exopolysaccharide and biofilm formation (*Δbps*), T3SS effector (BteA/BopC) delivery (*ΔbscN*), tracheal cytotoxin shedding (*ampG* knock-in), virulence factor expression fine-tuning (RisA D60N) and even production of pertactin (*Δprn*), respectively, were not any importantly affected in their capacity to colonize the nasal cavity of the immunocompromised MyD88 KO mouse. These mutants were also shed from the nasal cavity of infected mice and transmitted to co-housed recipient mice at very similar levels as the wild-type bacteria. It is, hence, plausible to assume that the disabled virulence factor functions, discovered in studies performed in immunocompetent mouse models, were required for full bacterial fitness and resistance to host immune defenses on the mucosa of fully immunocompetent hosts and not in the immunocompromised MyD88 KO mice. Particularly intriguing is the case of the pertactin mutant (*Δprn*). The autotransporter Prn protein of the outer bacterial membrane was initially described to function as an adhesin [2]. Later it was implicated in the resistance of *B*. *bronchiseptica* to neutrophil-mediated clearance in a mouse lung inflammation model [51]. Finally, it was recently shown to play an important role in triggering of shedding and transmission of the *B*. *bronchiseptica* bacteria from the nasal cavity of the TLR4-deficient C3H/HeJ mice [52]. However, we did not observe here any major contribution of PRN to the nasal colonization and shedding capacity of *B*. *pertussis* in the MyD88 KO mice. Only the transmission capacity of the *Δprn* mutant appeared to be slightly affected in that some co-housed mice did not acquire the infection from their index mates infected with the *Δprn* mutant. In line with a reduced role of PRN in transmission of the human pathogen *B*. *pertussis*, Prn-deficient *B*. *pertussis* isolates replaced the wild type bacteria under the aP vaccine pressure and are now spreading and predominantly accounting for pertussis cases in the populations of developed countries using the aP vaccine, such as in the USA. Hence, despite rather high sequence conservation, the role of Prn in the nasal colonization, shedding and transmission capacity of *B*. *pertussis* may differ substantially from its role played in the biology of *B*. *bronchiseptica* infections in rodents.

The most striking and significant phenotype of severally impaired transmission capacity was observed for the two mutants defective in production of the filamentous hemagglutinin (FhaB) and of the type I pilus called fimbria (Fim). It will be important to dissect at which step the FhaB and Fim play a key role in the infectious life cycle of *B*. *pertussis*, as a block at each of the adhesion, proliferation, shedding and new host mucosa colonization steps will prevent every other step. The FhaB and Fim proteins represent the key adhesive systems involved in *Bordetella* sp. binding to ciliated airway epithelial cells on which *B*. *pertussis* is typically found to form the cilia-interspersed microcolonies *in vivo* [85]. FhaB and Fim appear to synergize in playing an important role in *B*. *pertussis* resistance to innate immune defense [86]. As demonstrated for *B*. *bronchiseptica* infections of airways of its natural mouse host [2,87,88], the synergic action of FhaB with Fim appears to play a role in dampening of the inflammatory response to infection and reduces phagocyte infiltration onto infected mucosa. Consistent with that, the *ΔfhaB* and *Δfim B*. *pertussis* mutants exhibited a significantly decreased capacity to colonize the nasal cavity of the MyD88 KO mice, exhibiting a mean CFU count at least an order of magnitude lower than observed for wild-type bacteria and several other tested mutants. Moreover, most of the infected mice had several orders of magnitude lower nasal CFU loads (10^4^–10^6^) of *ΔfhaB* or *Δfim* mutant bacteria on day 7, compared to CFU counts reached by the parental strain, which consistently accumulated to 10^7^–5 x 10^8^ CFU per nasal cavity. Furthermore, the *ΔfhaB* and *Δfim* mutants were shed at about an order of magnitude lower numbers than parental bacteria, or most other individual gene mutants. Finally, the *ΔfhaB* and *Δfim* mutants failed to transmit and establish a productive infection in co-housed recipient mice at all, despite a close contact to infected index mice. These results strongly suggest that the concerted FhaB plus Fim-mediated adhesion to ciliated airway epithelial cells is central to the capacity of the pathogen to proliferate and persist within the nasal cavity, trigger nasal discharge, shed and productively infect new hosts. The FhaB and Fim adhesins thus represent key *B*. *pertussis* transmission factors at least in the newly established mouse model of *B*. *pertussis* transmission. This conclusion likely holds true also for *B*. *pertussis* transmission between humans, as by difference to massive expansion of pertactin-deficient isolates under the pressure of the acellular pertussis vaccine, FhaB-deficient isolates are isolated extremely rarely [89–91] and to the best of our knowledge, Fim-deficient clinical isolates of *B*. *pertussis* have not been reported at all. This indicates an essential function of FhaB and of Fim in *B*. *pertussis* colonization and transmission in humans. Therefore, identification of FhaB and Fim as key transmission factors lends credibility to the use of the immunocompromised mouse transmission model for identification of additional key colonization and transmission factors of the bacterium.

## Materials and methods

### Ethics statement

All animal experiments were approved by the Animal Welfare Committee of the Institute of Molecular Genetics of the Czech Academy of Sciences, v. v. i., in Prague, Czech Republic. Handling of animals was performed according to the *Guidelines for the Care and Use of Laboratory Animals*, the Act of the Czech National Assembly, Collection of Laws no. 246/1992. Permissions no. 75/2018 and 20/2020 were issued by the Animal Welfare Committee of the Institute of Molecular Genetics of the Czech Academy of Sciences in Prague.

### Bacterial strains and growth conditions

The *Escherichia coli* strain XL1-Blue was used throughout this work for plasmid construction and the *E*. *coli* strain SM10 λ pir was used for plasmid transfer into *B*. *pertussis* by bacterial conjugation. *E*. *coli* strains were cultivated at 37°C on Luria-Bertani (LB) agar medium or in LB broth. When appropriate, LB culture media were supplemented with ampicillin (pSS4245 plasmid transformants, 100 μg/ml). *B*. *pertussis* Tohama I was obtained from the Culture Collection of the Institute Pasteur in Paris under the catalogue No. CIP 81.32 and a spontaneous Str^R^ variant of it was isolated on Bordet-Gengou (BG) agar plates (Difco, Franklin Lakes, NJ, USA) supplemented with 1% glycerol, 15% defibrinated sheep blood (LabMediaServis, Jaromer, Czech Republic) and 100 μg/ml streptomycin. Bacterial cultures for intranasal infections were grown for 48 h on nutritionally enriched BG plates containing modified Stainer–Scholte medium [92] supplemented with 3 g/l of Casamino Acids and 1 g/l of heptakis (2.6-di-O-dimethyl) β-cyclodextrin (SSM) on top of the standard composition (BGX agar). Bacteria harvested from plates were suspended and diluted in prewarmed SSM to the density required for intranasal inoculation.

### Mutagenesis and deletions of *B*. *pertussis* genes

Mutant *B*. *pertussis* strains (Table 1) were constructed by allelic exchange as described previously [51] using the pSS4245 suicide vector (*ori*V, *Amp*R, *Str*R, *Km*R, *Ble*R, *Tet*R and an I-*Sce*I cleavage site for counterselection) kindly provided by Dr. Scott Stibitz (U.S. CBER, FDA). Briefly, fragments with ∼700 bp length of the upstream and downstream sequences of the open reading frames (ORFs) to be deleted or modified by point mutation were PCR-amplified by appropriate primers (listed in S1 Table) and inserted into the corresponding restriction site of pSS4245.

The mutated sequences were marked by silent mutations introducing unique restriction sites, which enabled straightforward verification of the presence of mutations by PCR analysis.

To construct double, triple or quintuple *B*. *pertussis* mutants, the mutations were successively introduced into the bacterial chromosome by allelic exchange one by one and were verified by restriction analysis and by resequencing of the corresponding PCR-amplified gene fragment.

Production of the respective proteins by the mutagenized strains was analyzed by Western blotting of whole bacterial cell lysates. The mouse monoclonal antibody (mAb) 9D4 was used to detect CyaA [93], the 63.1G9 mAb specific for the S1 subunit was used for detection of PT (Santa Cruz Biotechnology, Dallas, TX; diluted 1:500). Polyclonal mouse and rabbit sera raised against purified antigens were used to detect the FhaB, DNT, Vag8 and Prn proteins, respectively. Production of fimbriae was analyzed by ELISA using the mAb specific for Fim2 (Cat. No. 06/124, NIBSC, England), as previously described. [14,94].

### Mouse infection, bacterial shedding and transmission

Female and male BALB/c, C57BL/6J (Charles River, France), C57BL/6J MyD88 KO (KO, MyD88 null, B6.129P2(SJL)-Myd88^tm1.1Defr^/J, Jackson Laboratory, Bar Harbor, ME, USA), C57BL/6J TRIF^-/-^ (KO, C57BL/6J-*Ticam1*^*Lps2*^/J, Jackson Laboratory, Bar Harbor, ME, USA), C57BL/6J TRIF KO x MyD88 KO mice crossed in-house were bred and genotyped at the animal facility of the Institute of Molecular Genetics of the AS CR in Prague, Czech Republic.

The number of MyD88 KO animals per group varied in the different experiments due to slow breeding of the animals, which could not be purchased as large cohorts. Further, we aimed to respect the 3R rules and used only the numbers of mice needed for a robust demonstration of the observed colonization and transmission defects of *B*. *pertussis* mutants. For the WT *B*. *pertussis* strain 45 MyD88 KO animals have been used at the infection dose of 10^7^ CFU, as this group was always used as positive control in all MyD88 KO infection and transmission experiments with the various *B*. *pertussis* mutants. In such experiments, typically 2 to 3 animals per group were used and each infection/shedding/transmission experiment was repeated at least twice. When a phenotype of the mutant was observed, the number of animals per group was enlarged, as indicated in figure legends, to increase robustness and allow statistical significance calculations, where appropriate.

Wild type and mutant strains of *B*. *pertussis* CIP 81.32 were grown as described above on BGX plates for 48 h and the bacteria were then diluted in SSM to provide a challenge dose of 10^3^−10^9^ CFU in 5 μl. Mice were anesthetized by intraperitoneal (i.p.) injection of ketamine (80 mg/kg) and xylazine (8 mg/kg) in 0.9% saline. Five μl doses of bacteria were administered through nasal inhalation into both nostrils of the mouse. To determine viable CFUs of the *B*. *pertussis* inoculum, aliquots of the inoculum were diluted in PBS and plated on BG agar plates containing streptomycin (100 μg/ml) and grown at 37°C in a 5% CO_2_ atmosphere for 72 h to visualize hemolysis.

For determination of bacterial loads in mouse organs, mice were euthanized at indicated time points and the analyzed organs were aseptically removed. Lungs and trachea were homogenized in physiological saline with tissue grinders (Heidolph mechanical stirrers, Model RZR 2020, Merck, Darmstadt, Germany). Nasal cavities with turbinates were homogenized using an IKA Ultra Turrax T25 tissue homogenizer (Sigma-Aldrich, St. Louis, MO, USA) and cleared of bone debris by centrifugation for 10 min at 900 rpm (217 x g). Serial dilutions of homogenates were plated on BG agar supplemented with 15% defibrinated sheep blood and 100 μg/ml of streptomycin and the CFUs were counted after plate incubation for 4 days at 37°C.

Shedding of bacteria from the noses of the infected mice was quantified on days 4 and 7 by gentle tapping of infected mouse noses 4 times on the surface of a BG agar plate containing 15% sheep blood and supplemented with 100 μg/ml of streptomycin. 100 μl of PBS was added to each plate, the recovered bacteria were spread over the plates and grown at 37°C for 4 to 5 days prior to counting of hemolytic Str^R^ *B*. *pertussis* colony forming units (CFUs).

For transmission experiments, intranasally infected index (donor) mice were co-housed in the same cage with non-inoculated recipient mice at a 1:1 ratio for one week. On day 7 the nasal shedding of *B*. *pertussis* by the inoculated index mice was assessed before the index mice were sacrificed and bacterial loads in their nasal cavity were determined as described above. The recipient mice were kept for another week and nasal infection was assessed on day 14 after exposure to infected index mice.

### Histopathological analysis

Infection-triggered pathology was examined 7 days upon intranasal inoculation of MyD88 KO mice with 10^7^ CFU and C57BL/6J mice with 10^9^ CFU of *B*. *pertussis* Tohama I bacteria. Animals were sacrificed by cervical dislocation, heads were removed, cleaned from skin and hair and fixed in 4% Buffered Formaldehyde for 48 hours at 4°C. Decalcification was performed in Osteosoft (Merck #HC02057828) for 5 days at RT. The heads were next trimmed into 5 equal coronal sections and transferred into 70% EtOH for 24 hours at 4°C. Tissue samples were processed by Leica ASP6025 Tissue Processor using an automated protocol, embedded into FFPE blocks and two adjacent 2 μm thick sections were collected from each block onto SuperFrost+ slides. Hematoxylin-eosin (HE) staining and coverslipping was performed using Leica ST5020 Staining Machine and Leica CV5030 Coverslipper with an automated protocol. Following deparaffination, heat-induced antigen retrieval was performed in a pressure cooker at pH 8 in EDTA Buffer for 15 min at 110°C. The endogenous peroxidase activity was inactivated with 3% hydrogen peroxide in MeOH (v/v) for 15 min at room temperature and sections were blocked using 2% BSA (Roth, #0163.4) for 20 min at room temperature. Muc5AC detection with 1:200 diluted MUC5AC antibody (Invitrogen (MA5-12178) in Antibody Diluent RTU (Zytomed ZUC025-100) was performed at 4°C overnight, followed by decoration with HRP-Polymer anti-Mouse conjugate (Zytomed ZUC050-100) for 30 min at room temperature and visualized with DAB (DAKO K3468). The sections were counterstained with Alcian Blue solution for 30 min and hematoxylin (Biognost–HEMH-OT) for 2 min at room temperature. Macrophages were stained overnight at 4°C with Rabbit F4/80 antibody (Cell Signalling, #70076S) diluted 1:800 in Antibody Diluent RTU (Zytomed ZUC025-100) and revealed using HRP-Polymer anti-Rabbit RTU conjugate (Zytomed, #ZUC032-100) with DAB reaction and hematoxylin counterstaining. Polyclonal rabbit anti-*B*. *pertussis* serum diluted 1:2000 was applied overnight at 4°C in Antibody Diluent RTU (Zytomed ZUC025-100) and bacterial cells were decorated with HRP-Polymer anti-Rabbit RTU and DAB reaction, followed by hematoxylin counterstaining. NASDCL staining was performed according to protocol of the NASDCL Staining Kit (Sigma-Aldrich, #91C-1KT). PAS Alcian Blue staining was performed using an automated protocol (Ventana, #PAS-860-014 for PAS and #860–003 for Alcian Blue) using the BenchMark Special Stain Machine by Roche.

### Statistical analysis

Statistical analysis was carried out using the algorithms incorporated in the GraphPad Prism 7 package. One or two-way analysis of variance (ANOVA) followed by Dunnett’s multiple comparison test was used to analyze the statistical significance between groups. p values of less than 0.05 were considered statistically significant. * (p < 0.05); ** (p < 0.01); *** (p < 0.001); **** (p < 0.0001).

## Supporting information

S1 Fig Time course of *B*. *pertussis* shedding. Nares of MyD88 KO mice were inoculated with 10^9^ CFU of *B*. *pertussis* and nasal shedding was assessed at indicated time points by gentle tapping of the mouse noses 4 times on the surface of a BG agar plate supplemented with streptomycin (100 μg/ml) and spreading of deposited bacteria in 100 μl PBS. Shedding exceeding 10^3^ CFU per mouse nose/plate (dotted line) prevented more accurate CFU counting and its level was estimated from the density of the confluent *B*. *pertussis* lawns growing on BG agar and scored by the number of + signs indicating the estimated order of magnitude of shed bacterial numbers. Three mice per time points of 2 h and 28 days and 7–12 mice for other time points were used. Data for days 7, 14 and 21 were pooled from two independent experiments and the lines correspond to geometric means.
(TIF)

S2 Fig High level of *B*. *pertussis* shedding by the MyD88 KO mice does not enable infection of co-housed C57BL/6J mice. Index MyD88 KO mice inoculated into nares with 10^7^ CFU of *B*. *pertussis* cells were co-housed in the same cage in a 1:1 ratio with non-inoculated recipient C57BL/6J mice for 7 days before the index mice were withdrawn for determination of nasal shedding and nasal cavity bacterial loads. Reciprocally, index C57BL/6J mice inoculated into nares with 10^9^ CFU of *B*. *pertussis* cells were co-housed in the same cage in a 1:1 ratio with non-inoculated recipient MyD88 KO mice for 7 days before the index mice were withdrawn for determination of nasal shedding and nasal cavity bacterial loads. The co-housed recipient mice were kept for another week prior to determination of bacterial loads in their nasal cavities on day 14. Dots represent bacterial counts for individual mice. Horizontal bars indicate the geometric means. The number of mice used per challenge strain is given in brackets.
(TIF)

S1 Table List of PCR primers used for preparation of *B*. *pertussis* mutant strains. (DOCX)
