# Supplementary figures and images for: The Fim and FhaB adhesins play a crucial role in nasal cavity infection and Bordetella pertussis transmission in a novel mouse catarrhal infection model
Source: PLoS Pathog. 2022 Apr 8;18(4):e1010402. doi: 10.1371/journal.ppat.1010402 (PMC9020735; doi:10.1371/journal.ppat.1010402)

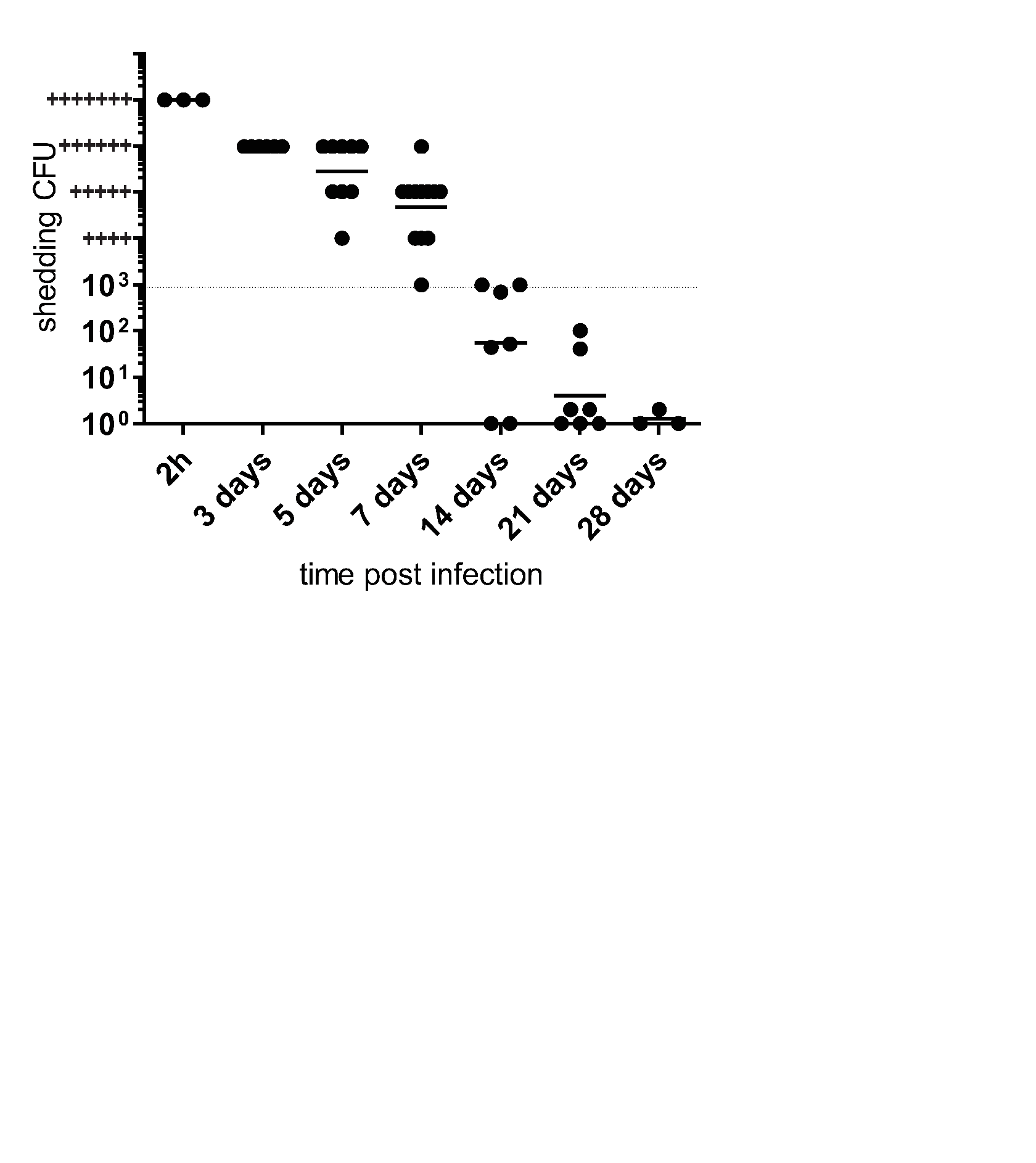

Supplement: S1 Fig — Nares of MyD88 KO mice were inoculated with 109 CFU of B. pertussis and nasal shedding was assessed at indicated time points by gentle tapping of the mouse noses 4 times on the surface of a BG agar plate supplemented with streptomycin (100 μg/ml) and spreading of deposited bacteria in 100 μl PBS. Shedding exceeding 103 CFU per mouse nose/plate (dotted line) prevented more accurate CFU counting and its level was estimated from the density of the confluent B. pertussis lawns growing on BG agar and scored by the number of + signs indicating the estimated order of magnitude of shed bacterial numbers. Three mice per time points of 2 h and 28 days and 7–12 mice for other time points were used. Data for days 7, 14 and 21 were pooled from two independent experiments and the lines correspond to geometric means. (TIF) [file ppat.1010402.s001.tif]

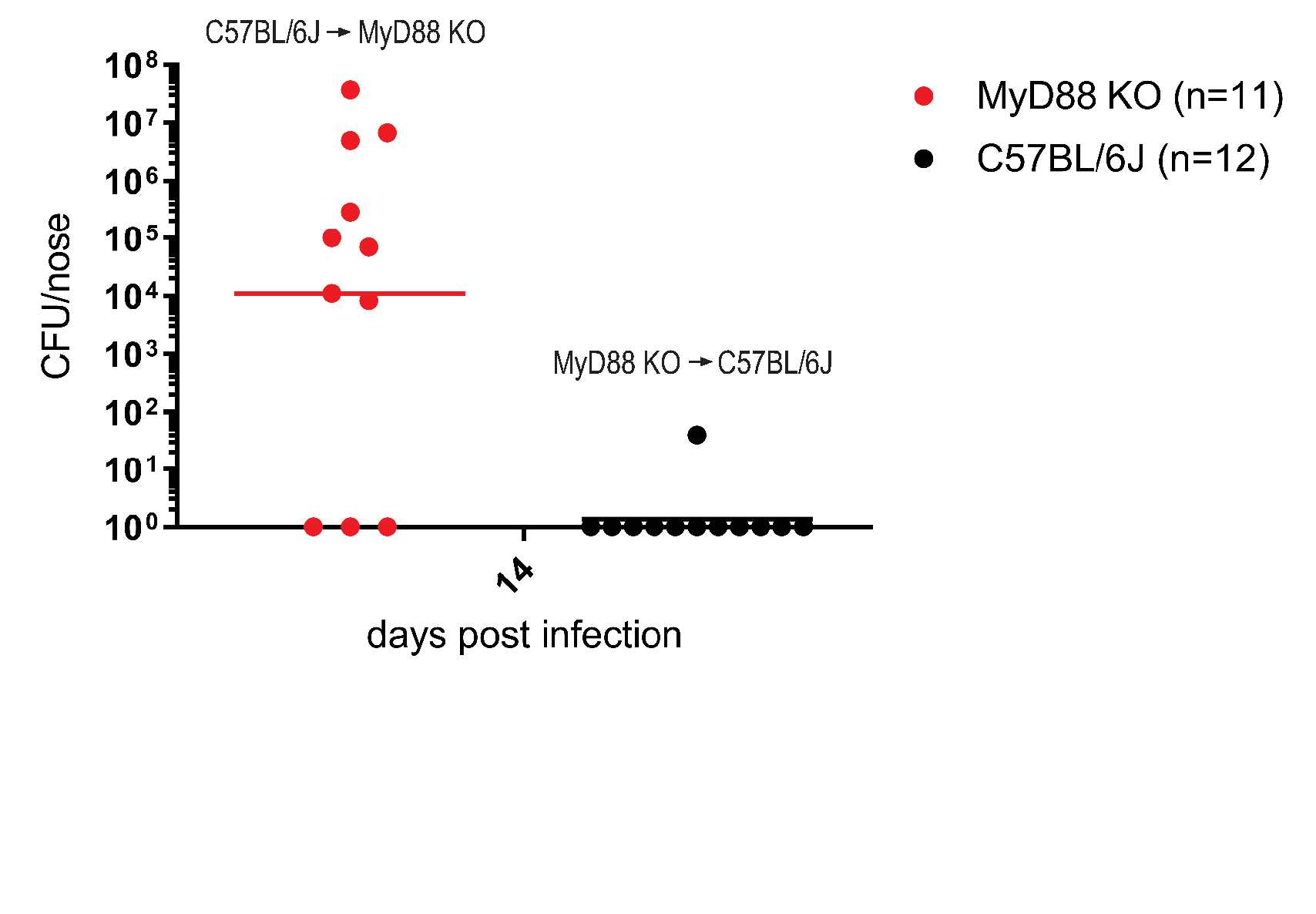

Supplement: S2 Fig — Index MyD88 KO mice inoculated into nares with 107 CFU of B. pertussis cells were co-housed in the same cage in a 1:1 ratio with non-inoculated recipient C57BL/6J mice for 7 days before the index mice were withdrawn for determination of nasal shedding and nasal cavity bacterial loads. Reciprocally, index C57BL/6J mice inoculated into nares with 109 CFU of B. pertussis cells were co-housed in the same cage in a 1:1 ratio with non-inoculated recipient MyD88 KO mice for 7 days before the index mice were withdrawn for determination of nasal shedding and nasal cavity bacterial loads. The co-housed recipient mice were kept for another week prior to determination of bacterial loads in their nasal cavities on day 14. Dots represent bacterial counts for individual mice. Horizontal bars indicate the geometric means. The number of mice used per challenge strain is given in brackets. (TIF) [file ppat.1010402.s002.tif]
