# Supplementary material for: The Fim and FhaB adhesins play a crucial role in nasal cavity infection and Bordetella pertussis transmission in a novel mouse catarrhal infection model
Source: PLoS Pathog. 2022 Apr 8;18(4):e1010402. doi: 10.1371/journal.ppat.1010402 (PMC9020735; doi:10.1371/journal.ppat.1010402)
Supplement: S1 Table — (DOCX) [file ppat.1010402.s003.docx]

**S1 Table.**

**List of PCR primers used for preparation of *B. pertussis* mutant strains.**

The mutant strain and primer sequence (5´- 3´) are provided.

| **Mutant strain /**  **mutation** | **Primer sequence for / rev** |
| --- | --- |
| *Bp* ΔbscN | BscNh1f ctcgACTAGTctggtgctgtccatgctcgaaatcc  BscNh1r TTAggatccGAGCTCctcgagCATgcccctatccgcgcc  BscNh2f ATGctcgagGAGCTCggatccTAAtggacctggaaagcctgcttgc  BscNh2r tcagGAATTCcagttcttccttcgctttctcctgttcc |
| *Bp* Δbps | Bps delta SpeI for CTACTAGTGACCATGGTTTCGACGGGGTC  Bps delta StuI rev CTAGGCCTTGAGCGCTCAGGCGAGCGC  Bps delta StuI for CTAGGCCTTCATAGGACAGCGTTCTACGAA  Bps delta SacI rev CTGAGCTCGTACGGCAAGTGTAAGGGCATA |
| *Bp* Δdnt | dnt NotI for CTGCGGCCGCAGACCGGGCAGACGATCAAC  dnt SpeI rev CTACTAGTATCCACGTTTGTTTGCCCCTG  dnt SpeI for CTACTAGTGTCTGAATCGCATTCCGCGAA  dnt EcoRI rev CTGAATTCGCCGATGACCATCATTGCCCG |
| *Bp* Δvag8 | Vag8 up for CCTATGCTAGGGCGGCCGCAGATAGGTCGGGTGTGGAAGC  Vag8 up rev GCCGGCGGCGCGCGGTCACATCTCAACACCTCTTGGCTAG  Vag8 down for CTAGCCAAGAGGTGTTGAGATGTGACCGCGCGCCGCCGGC  Vag8 down rev AGGACGCGTGGATCCGAATTCCTGGGCCATCATGACCAAC |
| *Bp* ΔlgmB | ArnT NotI for CTGCGGCCGCACGGCGACGCCGTCATCTGTC  ArnT SpeI rev GAACTAGTCATTGGGAACGCGCCTTGGCC  ArnT SpeI for CTACTAGTTAGCGACGAATGTCAGGAAGGC  ArnT BamHI rev GAGGATCCAGCTGGCCGGCGTAG |
| *Bp* ΔcyaA | cyaA delta SpeI for CTACTAGTGTCCATTAGGGCCCAGCTGC  cyaA delta StuI rev CTAGGCCTCATGTGGATCTGTCGATAAGTAG  cyaA delta StuI for CTAGGCCTTGAAGCGCCGTGAATCACGG  cyaA delta SacI rev CTGAGCTCCTGCGATGACATTGAGCGTTTC |
| *Bp* cyaA*-*AC^-^ | Act mut for GACGGCGGATGGATCCATCGACATGTTC  Act mut rev GAACATGTCGATGGATCCATCCGCCGTC  Act NotI for CTGCGGCCGCATGCAGCAATCGCATCAGGCTG  Act BamHI rev CTGGATCCCGAGAACTTCGAACGTCCGCTC |
| *Bp* RisA D60N | RisA SpeI for GTACTAGTCGGGCCATTAGGTCAACTGC  RisA D60N rev GGTTCTCAACCTGATGCTCCCGGGCGAG  RisA D60N for CTCGCCCGGGAGCATCAGGTTGAGAACC  RisA BamHI rev CGGGATCCCCAGCCCCAG |
| *Bp* ΔTCT | Bp pseudo NotI for CTGCGGCCGCAATTGGCGCCGCGCACCACC  Bp pseudo SpeI rev CTACTAGTCCGATGCGTCCGCCGTGCAG  ampG SpeIE for CTACTAGTCATCGCTACCGCGCAGAATGG  ampG SacIE rev CTGAGCTCTTACGTCAGATGCGTTTTTCGTAG  Bp pseudo SacI for CTGAGCTCCCTGATGGGCACCTCGGCCAC  Bp pseudo BamHI rev CTGGATCCGCGTCACGGTGTTCCTGGTCG |
| *Bp* Δprn | Prn NotI for CTGCGGCCGCAGCAAGACTGCGAGCTGCTG  Prn SpeI rev CTACTAGTCATTGGATGCCAGGTGGAGAG  Prn SpeI for CTACTAGTTAAAGCGAGGAGGGTCTATCC  Prn BamHI rev CTGGATCCTGGTCAGCGAGGGCGTGC |
| *Bp* Δptx | Ptx delta SpeI for CTACTAGTGCGGTGCTGGAACATATCC  Ptx delta SacI rev CTGAGCTCCATCCCGTCTTCCCCTCTG  Ptx delta SacI for CTGAGCTCTGAGCCGCCGGCTCGGATC  Ptx delta BamHI rev CTGGATCCCAGCGGCGCATAGACGGTAC |
| *Bp* ptx R9K E129G | ptxS1 mutRK for CCACCGTGTACAAGTATGACTCCCG  ptxS1 mutRK rev CGGGAGTCATACTTGTACACGGTGG  ptxS1 mutEG for CTACCAGAGCGGATATCTGGCACAC  ptxS1 mutEG rev GTGTGCCAGATATCCGCTCTGGTAG  ptxS1 XhoI for GGTCTATCTCGAGCATCGCATGCAG  ptxS1 XhoI rev CTGCATGCGATGCTCGAGATAGACC  ptxS1 NotI for CTGCGGCCGCCCTGGTTCGCCGTCATGGCC  ptxS1 BamHI rev CTGGATCCGACCAGCCGCGCGTCACATG |
| *Bp* Δfim | Fim SpeI for GCACTAGTGCTGAGGTGGGCAAGGCTA  Fim XhoI rev ACCTCGAGAACCCTGCGAATCCCCATG  Fim XhoI for TTCTCGAGGTATGACTGACGCAACGAAC  Fim EcoRI rev CCGAATTCTTGATGCGGCCCCACTCC |
| *Bp* ΔfhaB | Fha NotI for CTGCGGCCGCGGCATTGATGACCTCGTGCAG  Fha SpeI rev GAACTAGTCGTGTTCATATTCCGACCAGC  Fha SpeI for CTACTAGTAACAAATAGGTAGTCGCGGCCTG  Fha BamHI rev GAGGATCCCATGCCGCCTTGCCGCTTTAC |
| *Bp* ΔfhaB-fim | Del FhaFimNotI for CTGCGGCCGCGGCATTGATGACCTCGTGCAG  Del FhaFimSpeI rev CTACTAGTCGTGTTCATATTCCGACCAGC  Del FhaFimSpeIfor CTACTAGTTTGAACTATGACTGACGCAACG  Del FhaFimBamHI rev CTGGATCCCGTTGTACTTGTAGCGGCC |
